# FOntCell: Fusion of Ontologies of Cells

**DOI:** 10.3389/fcell.2021.562908

**Published:** 2021-02-11

**Authors:** Javier Cabau-Laporta, Alex M. Ascensión, Mikel Arrospide-Elgarresta, Daniela Gerovska, Marcos J. Araúzo-Bravo

**Affiliations:** ^1^Computational Biology and Systems Biomedicine Group, Biodonostia Health Research Institute, San Sebastián, Spain; ^2^Computational Biomedicine Data Analysis Platform, Biodonostia Health Research Institute, San Sebastián, Spain; ^3^Basque Foundation for Science (IKERBASQUE), Bilbao, Spain; ^4^Centro de Investigación Biomédica en Red (CIBER) of Frailty and Healthy Aging (CIBERfes), Madrid, Spain; ^5^TransBioNet Thematic Network of Excellence for Transitional Bioinformatics, Barcelona Supercomputing Center, Barcelona, Spain; ^6^Computational Biology and Bioinformatics, Department Cell and Developmental Biology Max Planck Institute for Molecular Biomedicine, Münster, Germany

**Keywords:** ontology alignment, ontology merging, automatic ontology merging, cell ontology, Human Cell Atlas (HCA), Ontology Alignment Evaluation Initiative (OAEI)

## Abstract

High-throughput cell-data technologies such as single-cell RNA-seq create a demand for algorithms for automatic cell classification and characterization. There exist several cell classification ontologies with complementary information. However, one needs to merge them to synergistically combine their information. The main difficulty in merging is to match the ontologies since they use different naming conventions. Therefore, we developed an algorithm that merges ontologies by integrating the name matching between class label names with the structure mapping between the ontology elements based on graph convolution. Since the structure mapping is a time consuming process, we designed two methods to perform the graph convolution: vectorial structure matching and constraint-based structure matching. To perform the vectorial structure matching, we designed a general method to calculate the similarities between vectors of different lengths for different metrics. Additionally, we adapted the slower Blondel method to work for structure matching. We implemented our algorithms into FOntCell, a software module in Python for efficient automatic parallel-computed merging/fusion of ontologies in the same or similar knowledge domains. FOntCell can unify dispersed knowledge from one domain into a unique ontology in OWL format and iteratively reuse it to continuously adapt ontologies with new data endlessly produced by data-driven classification methods, such as of the Human Cell Atlas. To navigate easily across the merged ontologies, it generates HTML files with tabulated and graphic summaries, and interactive circular Directed Acyclic Graphs. We used FOntCell to merge the CELDA, LifeMap and LungMAP Human Anatomy cell ontologies into a comprehensive cell ontology. We compared FOntCell with tools used for the alignment of mouse and human anatomy ontologies task proposed by the Ontology Alignment Evaluation Initiative (OAEI) and found that the F_β_ alignment accuracies of FOntCell are above the geometric mean of the other tools; more importantly, it outperforms significantly the best OAEI tools in cell ontology alignment in terms of F_β_ alignment accuracies.

## Introduction

Precision biomedicine technologies produce overgrowing quantities of information from of high throughput data from finer-grained biomedical samples reaching single-cell (Hwang et al., 2018) and subcellular (Grindberg et al., 2013) levels that allow to discover new cell types (Boldog et al., 2018; Gerovska and Araúzo-Bravo, 2019; Sas et al., 2020). This increasingly precise cell data render existing cell classification systems obsolete and create the demand for automatic comprehensive data-driven cell classification methods. Among the structures to classify knowledge domain items are the ontologies; they can be defined in several ways depending on the context of use (Busse et al., 2015). In information science, an ontology is defined as a seven-tuple, O:= {L, C, R, F, G, T, A}, where L:=LC ⋃ LR is a lexicon of concepts LC and relations LR; C is a set of concepts; R is a set of binary relations on C; F and G are functions connecting symbols F: LC → C, G: LR → R; T is a taxonomy for the partial ordering of C, T(C_*i*_, C_*j*_), and A is a set of axioms with elements C and R (Busse et al., 2015). A critical question in the design of an ontology is the level of detail covered by the ontology. Thus, different ontologies of the same knowledge domain use different conceptualizations to obtain the desired level of granularity. In the case of cell ontologies, there are several cell type classifications in various formats; the most frequently used being the Web Ontology Language (OWL) (Smith et al., 2004) format, that encompass the vast majority of Open Biomedical Ontologies (OBO) Foundry (Smith et al., 2007) ontologies.

Cell classification relies on human data curation, however the growing number of discovered new cell types boosted by high throughput data generation such as single cell RNA-Seq and international research initiatives such as the Human Cell Atlas (HCA) (Rozenblatt-Rosen et al., 2017) creates a necessity to develop automatic computational methods that assist the creation of cell ontologies and the classification of these new cells as branches of existing cell ontologies (Osumi-Sutherland, 2017). New cell ontologies can be created by reusing and merging the information dispersed in multiple cell ontologies. Before merging two ontologies, it is necessary to find the correspondences between their concepts in a process named ontology alignment or matching. There exist numerous tools for the alignment and merging of ontologies (Table 1). The majority of them are semi-automatic since they require an initial input and some intermediate user inputs for performing the alignment; some tools focus only on the alignment.

**Table 1:** Tools for the alignment and merging of ontologies and their features, adapted from Table 9 from Lambrix and Tan (2006).

**Tool**	**Linguistic**	**Structure**	**Constraints**	**Auxiliary**	**Automatic**	**Merging**	**References**
ArtGen	Name	Parents, children		WordNet	Semi or fully		Mitra and Wiederhold, 2002
ASCO	Name, label, description	Parents, children, siblings, path from root		WordNet	Fully		Le et al., 2004
Chimaera	Name	Parents, children			Semi	Merging	McGuinness et al., 2000
FCA-Merge	Name				Semi	Merging	Stumme and Maedche, 2001
FOAM	Name, label	Parents, children	Equivalence		Semi		Ehrig and Staab, 2004
GLUE	Name				Semi		Doan et al., 2004
HCONE	Name	Neighborhood		WordNet	Semi	Merging	Kotis and Vouros, 2004
IF-Map		Parents, children		Reference ontology	Semi		Kalfoglou and Schorlemmer, 2003
iMapper			Domain, range	WordNet	Semi		Su et al., 2004
Onto Mapper	Name	Parents, children			Semi		Prasad et al., 2002
Anchor-PROMPT	Name	Direct graphs			Semi	Merging	Noy and Musen, 2000
SAMBO	Name, synonym	Is-a a part-of, descendants & ancestors		WordNet UMLS	Semi	Merging	Lambrix and Tan, 2006
S-Match	Label				Fully		Giunchiglia et al., 2004
AML	Label, instances	Direct graph, logical repair algorithm		WordNet	Fully		Faria et al., 2013
LogMap	Label, name	Linguistic alignment, principle of locality		WordNet, UMLS-lexicon	Semi or fully		Jiménez-Ruiz and Cuenca Grau, 2011
AGM	Name, label	Graphs			Semi or fully		Lütke, 2019
ALIN	Label			Wordnet	Semi		da Silva et al., 2019
DOME	Label	doc2vec			Fully		Hertling and Paulheim, 2019
FCAMap-KG	Label, synonym	Part-of			Semi or fully		Zhao et al., 2018
Lily	Name, label	Direct graphs			Semi or fully		Wang and Xu, 2008
LogMapBio	Label, name	Linguistic alignment, principle of locality		WordNet, UMLS-lexicon, BioPortal	Semi or fully		Jiménez-Ruiz, 2019
LogMapLite	Label, name			WordNet, UMLS-lexicon	Semi or fully		Jiménez-Ruiz, 2019
POMAP++	Lame, label		Ontology attribute, linguistic match		Semi		Laadhar et al., 2017
FOntCell	Label, synonym	Direct graphs, attribute relation	Ontology attribute, linguistic match		Fully	Merging	This work

*Tool: Algorithm name. Linguistic: type of data used by the linguistic based method. Structure: type of data used by the structure based method. Constraints: type of data used to perform a constraint-based alignment. Auxiliary: external tool used to improve the alignment. Automatic: automation level; fully, if only is required an initial input; semi, if some additional intermediate user inputs are required. Merging: if merging is done in addition to the alignment. Tools like ATOM (Raunich and Rahm, 2011) are omitted since they are ontology merging methods that require a mapping as input and they do not use alignment methods*.

In order to minimize human supervision of the ontology alignment and automate the ontology merging, we developed an algorithm and implemented it into FOntCell, a software package in Python for automatic merging of ontologies. We applied FOntCell to create a new more comprehensive and fine-grained ontology of the cellular development by merging cell ontologies giving rise to all cell types of the human body.

There are multiple ontologies with biomedical information (genomics, proteomics, and anatomy) (Lambrix et al., 2007). Two of the largest cell ontologies are CELDA (Seltmann et al., 2013) and LifeMap (Edgar et al., 2013). CELDA integrates information about gene expression, localization, development and anatomy of *in vivo* and *in vitro* human and mouse cells, as well as cell development. Therefore, we focused on the “development” annotation information of CELDA stored in the fields CL (Cell Ontology) (Bard et al., 2005), CLO (Cell Line Ontology) (Sarntivijai et al., 2014) and EFO (Experimental Factor Ontology) (Malone et al., 2010). Another important repository for cell information is LifeMap (Edgar et al., 2013); which includes cell type and gene expression annotations of cells in different stages of embryonic development. LifeMap provides contrasted data and enough cell types to be synergistically merged with CELDA, as each might have cell types missing in the other. A hurdle in the merging CELDA and LifeMap is their different labeling systems. Also there are more specific ontologies such as the Cell Ontology for Human Lung Maturation [LungMap Human Anatomy (LMHA)] that is a specific ontology of cells for lung development. Theseontologies use different labels for the same cell type and simple word matching cannot find equivalences. Therefore, it is necessary to align the ontologies (Lambrix and Tan, 2008), i.e., identify the classes of one ontology equivalent to the classes of the other ontology. We developed an algorithm that can find equivalence between two classes from two ontologies, taking into account not only the class labeling but also the internal structure of the ontologies.

## Materials and Methods

**Figure 1:**
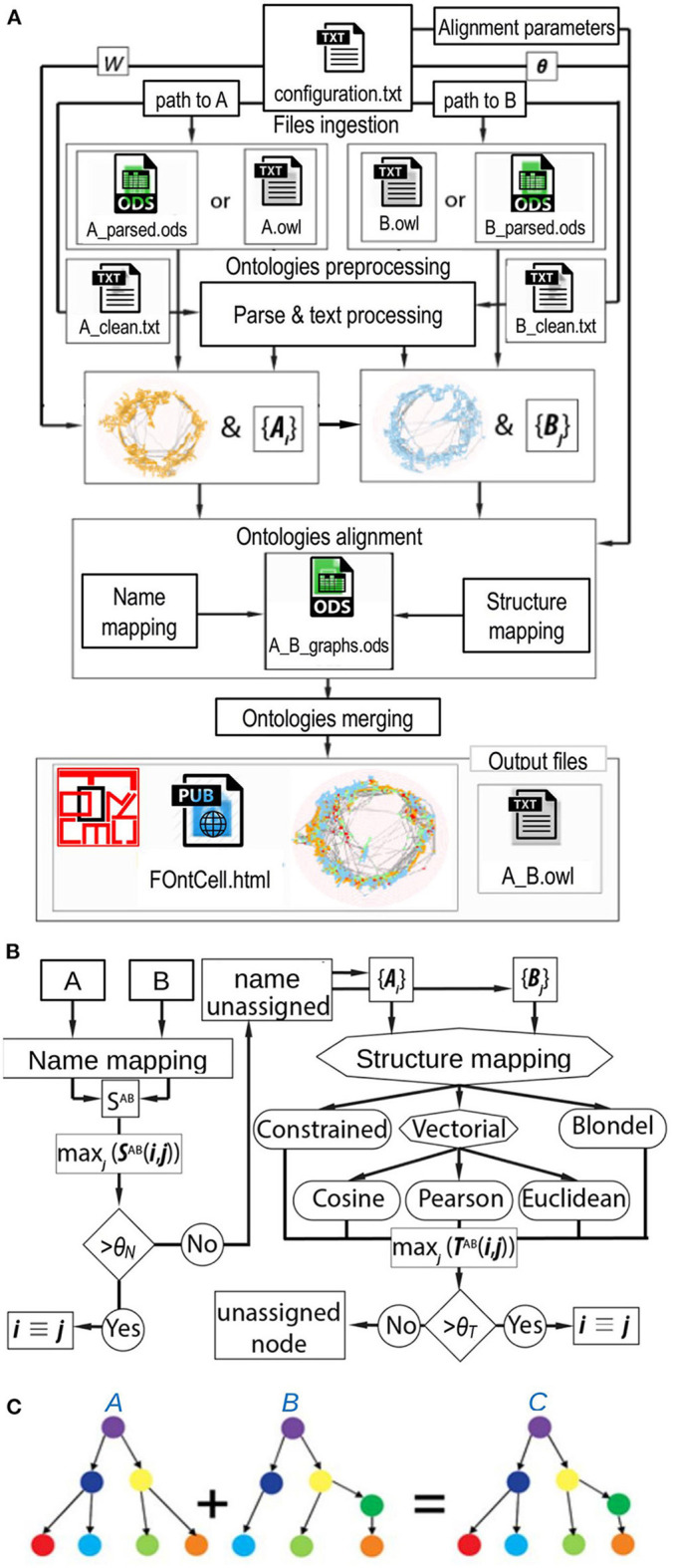
FOntCell algorithm. **(A)** FOntCell software flux diagram with the main functionalities of files ingestion, ontologies preprocessing, ontology parsing, ontologies alignment, ontologies merging, and generation of output files. Together with the ontology files, the user feeds the alignment parameters: *W*, window length, and the similarities threshold vector θ = {θ_*N*_, θ_*T*_, θ_*LN*_}. **(B)** Flux diagram of the FOntCell alignment algorithm combining the name mapping (left) and the structure mapping (right) using five alternative mapping methods. {*A*_*i*_} and {*B*_*j*_} denote the sets of subgraphs around nodes *i* and *j* of ontology A and B, respectively. The rhombi and octagons mark two or three alternative decisions, respectively. **(C)** Conceptual example of merging of ontologies. For merging two ontologies A and B into an ontology C, FOntCell aligns equivalent classes between A and B, and then merges the non-common relations that branch from the equivalent classes. Equivalent classes are marked with same colors in the two ontologies A and B.

The main steps for ontology merging implemented in FOntCell are file ingestion, ontology parsing, ontology pre-processing, alignment, and merging. The internal relations of the ontologies that will be merged and the alignment parameters are specified in a configuration file (Figure 1A). The format of the configuration file is described in detail in the Supplementary Material. An instance of the configuration file for the merging of CELDA and LifeMap and their result with LMHA are also provided in the Supplementary Material. The equivalent classes are detected by a combination of name matching and graph-topology/structure similarity matching (Figure 1B). The merging works through expansion of the non-common relationships/edges branching from the equivalent classes. FOntCell searches for similar (to match them) and different (to append them during the merging) classes (Figure 1C).

### Ontology Parsing

The parsing of the input ontologies generates two two-column matrices, *A2* and *B2*, each one with a number of rows equal to the number of class relations in the respective ontology. The first column contains the name of each class, and the second column, the name of one of its children. The matrices *A2* and *B2* are needed for the structure-mapping. FOntCell can merge any two ontologies in an .owl file in an OWL format, or in .ods files in parent-child relationship format compatible with the pyexcel-ods Python module. Additionally, FOntCell can read as input the matrices *A2* and *B2* in tabulated text files.

### Ontology Pre-processing

FOntCell can merge ontologies that share some classes and knowledge domain; however, our main interest is to apply FOntCell to augment cell ontologies by merging known cell ontologies. Different ontologies use diverse description formats and data structures; additionally, some ontologies are ill-formed with redundant (duplicates) or missing relationships (disconnected branches). FOntCell robustly merges such ill-formed ontologies. However, we implemented an optional pre-processing functionality allowing to repair the input ontologies, select the data relation type (instances, children, parents) used as an input argument, and edit labels to modify the original ontology relationships by addition, deletion and/or merging of classes and relationships.

The pre-processing stage of FOntCell is implemented as an “Automatic ontology editor” that takes as an input an ontology edition .txt file (format given in the Supplementary Material) describing the pre-processing modifications of the pre-processed ontology. FOntCell uses such description to modify the class names and/or rewire the ontology. Among the implemented pre-processing functions of the “Automatic ontology editor” are: (a) Selection of classes by their name, i.e., select classes including a certain word. (b) De-selection of classes to exclude classes from the resulting ontology. (c) Connection of two previously unrelated classes. (d) Merging of two classes with the resulting class “inheriting” the ID of one of the classes, and the relations and attributes of both classes. (e) Class label preconditioning to edit the class names and class synonyms, and eliminate general terms such as “the,” “cell,” “cells,” “human,” “mouse” or “-.” The “Automatic ontology editor” was used to pre-process the ontologies in this work as follows.

#### CELDA Pre-processing

CELDA uses information from other ontologies and its original structure is disconnected; split into several trees, and contains information related to tissues, immortal cell lines, species, etc. Since we are only interested in the developmental cell type information, we need to parse to cell types. Thus, to generate a connected graph of cell development from CELDA, we used the “Automatic ontology editor” to: (1) Introduce new relationships between classes to eliminate discontinuities, some of which are due to the word “human” or “mouse” in the name of their classes. (2) Merge duplicated classes due to the same cell type appearing simultaneously from “human” and “mouse”. (3) Eliminate the classes associated to immortalized cell lines selected as they contained “human” or “mouse” in the name, label or synonyms.

#### LifeMap Pre-processing

The pre-processing of LifeMap is required since LifeMap is an online database in non-OWL format, where its information about cell development is available at the LifeMap website repository (Edgar et al., 2013). We automatically searched the LifeMap website and obtained all the information related to cell name and synonyms, development hierarchy and cell localization and saved it to a two-column matrix file in .ods format. We used the “Automatic ontology editor” to perform string normalization by eliminating the symbols {“-,” “/,” “,”} and the words {“cell,” “cells,” “human”) from cell names and synonyms.

#### LMHA Pre-processing

LMHA presents in its “natural” ontology a series of cell types from the lung, but without a direct relationship in the cell development of the tissue itself. We used the “Automatic ontology editor” to: (1) Remove classes that do not provide information about specific cell types such as the “immune cell” or “cell type” classes. (2) Provide some new relations and synonyms.

The sizes of the processed ontologies before and after preprocessing and shown in Table 2. The same pre-processed ontologies files were used in the rest of the work by all the merging ontology tools.

**Table 2:** Sizes of the processed ontologies before and after preprocessing.

	**Before pre-processing**		**After pre-processing**	
	**#Classes**	**#Relations**	**#Classes**	**#Relations**
CELDA	15,439	203,058	841	966
LifeMap	796	924	796	924
CELDA + LifeMap	1,408	1,855	–	–
LMHA	80	130	45	65
(CELDA + LifeMap) + LMHA	1,437	1,919	–	–

### Calculation of the Name Mapping Matrix

Before performing the intra-ontology name matching, FOntCell processes the string of each label class of each ontology. Among other string processing tasks, FOntCell performs string normalization, removes mismatching words, splits words, selects substrings, selects only the class name, or optionally uses lists of synonyms representing variation of the class names. Next, it builds a name mapping matrix *S*^AB^(*a* × *b*), where *a* and *b* are the number of classes of ontologies A and B, respectively. Each element *S*^AB^(*i,j*) is a measurement of the similarity between the labels of class *i* from ontology A and class *j* from ontology B. Additionally, the user can trigger a FOntCell option that takes into account synonym attributes for the calculation of the name mapping matrix *S*^AB^.

In the simplest case of not activating the option of using synonyms, to measure the similarity between each class label of two ontologies A and B, FOntCell builds a name mapping matrix, *S*^AB^, based on the Levenshtein metric (Levenshtein, 1966), which measures the minimum number of insertions, deletions and necessary replacements to make two strings equal. To obtain the similarity in the range [0, 1], we use the opposite of the scaled Levenshtein metric:

(1) $$S^{AB}\left(i,j\right)=1-\frac{\text{lev}\left({\text{Label}}_{\text{i}}^{\text{A}},{\text{Label}}_{\text{j}}^{\text{B}}\right)}{\max\left(\left|{\text{Label}}_{\text{i}}^{\text{A}}\right|,\left|{\text{Label}}_{\text{j}}^{\text{B}}\right|\right)}$$

where *lev* is the Levenshtein distance between two strings. For two strings *a* and *b* of lengths |*a*| and |*b*|, respectively, the Levenshtein distance *lev*(|*a*|,|*b*|) is:

(2) $$lev\left(\left|a\right|,\left|b\right|\right)=\{\begin{array}{cc} max\left(i,j\right) & if\;\min\left(i,j\right)=0,\\ \min\{\begin{array}{c} lev\left(i-1,j\right)+1\\ lev\left(i,j-1\right)+1\\ lev\left(i-1,j-1\right)+1_{\left(a_{i}\neq b_{j}\right)} \end{array}\; & otherwise. \end{array}$$

where 1_(*ai≠ bj*)_ is the indicator function equal to 0 when *a_i_* = *b*_j_, and equal to 1 otherwise, and *lev*(*i,j*) is the distance between the first *i* characters of *a* and the first *j* characters of *b*. ${Label}_{i}^{A}$ and ${Label}_{i}^{B}$ are the labels of the class *i* and *j* of the ontologies A and B, respectively, and | | is the length of the string. Applying the pairwise Equation (1) for each stripped label class *i* of A, and the stripped label class *j* of B, FOntCell builds the name mapping matrix *S*^AB^ between A and B.

In the case of selecting the option to use the classes synonyms, the similarity between two classes *i, j* is calculated using the lists of synonyms, {*i*}∈A and {*j*}∈B that include also the principal label of the class. With these lists is calculated a name matching matrix *S*^{*i*}{*j*}^ (|{*i*}| × |{*j*}|) between each synonym of class list {*i*} and each synonym of class list {*j*} based on the Levenshtein distance (Equation 2), |{*i*}| and |{*j*}| are the lengths of the lists {*i*} and {*j*}, respectively. Finally, the highest score of *S*^{i}^^{*j*}^ as the matching between the two classes is taken to be used in the final name mapping matrix *S*^AB^(*i,j*) = max *S*^{i}^^{*j*}^. FOntCell considers that two labels have a name matching, if their score given by Equation (1) is greater than a name score threshold θ_*N*_ (default 0.85).

### Calculation of the Structure Mapping Matrix

Not all classes of an ontology are identifiable as classes of the other ontology by name mapping. For example, in the CELDA and LifeMap merging, when using only the name mapping, approximately 60% of the classes from CELDA are initially not assigned to LifeMap. One of the functionalities of FOntCell is to recognize matches between two ontologies and merge them into a unique class, i.e., two labels of two classes having very different name labeling but corresponding to the same concept. FOntCell discovers synonymous classes between two ontologies using structure mapping, i.e., two classes match if the subgraphs corresponding to their descendants have similar structures.

**Figure 2:**
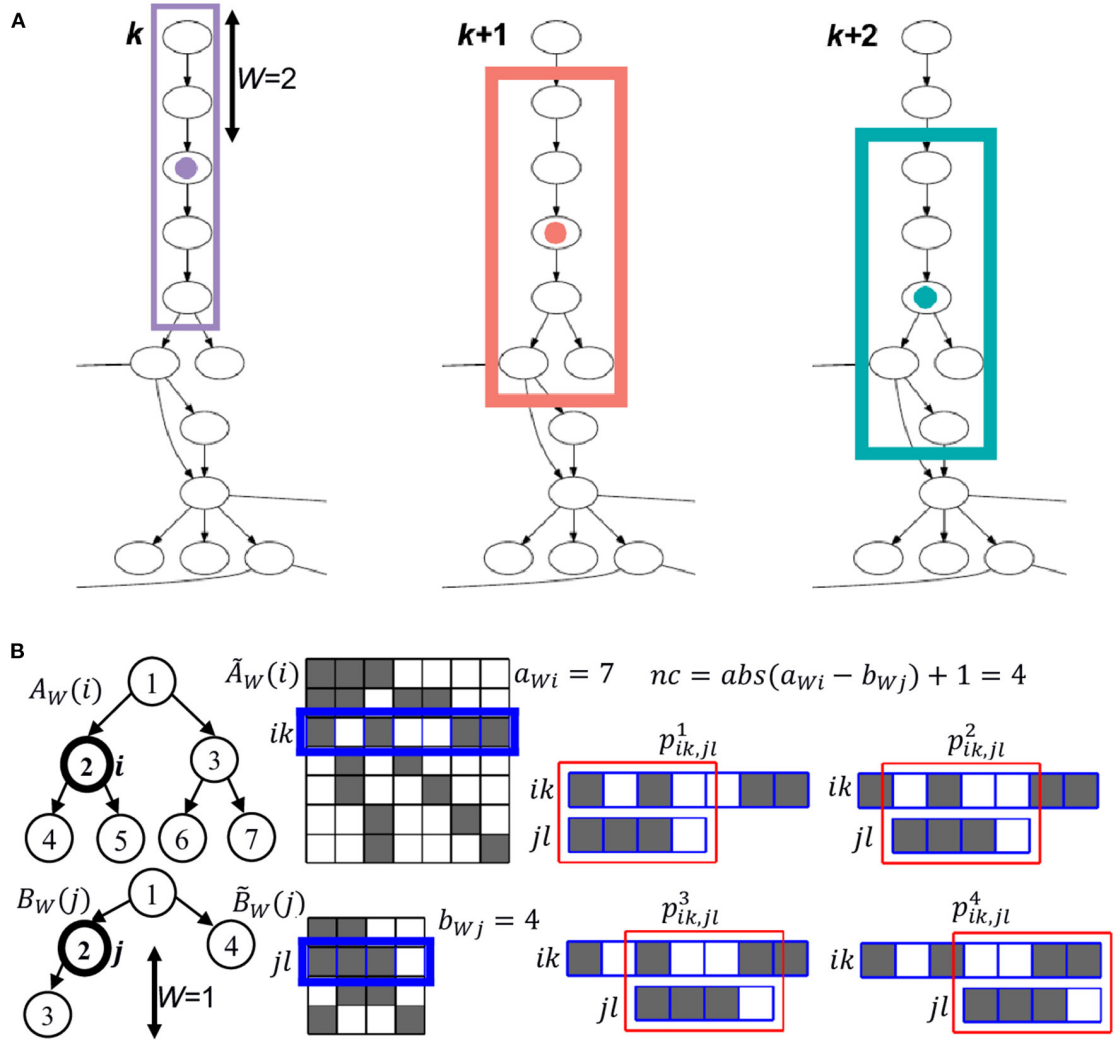
Convolutional graph matching of FOntCell. **(A)** Example of three consecutive steps of the sliding window of length *W* = 2 used in the calculation of the structure convolutional matching. For each central (generator) node, marked with a colored circle, the nodes involved in the calculation of the structure convolutional matching are framed with a rectangle of the same color as its corresponding central node. **(B)** Example of graph convolution, for a sliding window of length *W* = 1, between two subgraphs *A*_*W*_(*i*) and *B*_*W*_(*j*) (left) with generator nodes *i* and *j*, adjacency matrices ${\tilde{A}}_{W}\left(i\right)$ and ${\tilde{B}}_{W}\left(j\right)$ (center), and number of nodes *a*_*Wi*_ = 7 and *b*_*Wj*_ = 4, respectively. The connected nodes are represented by dark cells in the adjacency matrices. For each row *k* of ${\tilde{A}}_{W}\left(i\right)$, and *l* of ${\tilde{B}}_{W}\left(j\right)$, a vectorial convolution is calculated. The step for the rows *k* = 3 of ${\tilde{A}}_{W}\left(i\right)$, and *l* = 2 of ${\tilde{B}}_{W}\left(j\right),\;$is marked in blue as an example. The *nc* = abs(a_*Wj*_ *- b*_*Wi*_) + 1 = 4 sliding windows of the shorter row *jl* of ${\tilde{B}}_{W}\left(j\right)$ over the longer row *ik* of ${\tilde{A}}_{W}\left(i\right)$ are marked in red (right), and the respective *nc* convolution similarities $p_{ik,jl}^{c}$ for each slide *c* are calculated using one of the metrics *M* = {1 - cosine, Euclidean, 1 - Pearson}.

To relate the nodes of two ontologies, FOntCell extracts a local subgraph centered on each node, that we name generator nodes *i* from ontology A and generator nodes *j* from ontology B. The set of all subgraphs from the ontologies A and B are designated as {A} and {B}, respectively. The subgraphs extracted from the generators nodes *i* and *j* are denoted {*i*} and {*j*}, respectively. The size of these subgraphs is given by the parameter *W*, which indicates the number of upstream and downstream relationships from the generator node that FOntCell takes to create the subgraphs (Figure 2A).

FOntCell measures the structure similarity mapping between two graphs using different methods to build a structure mapping matrix *T*^AB^(*a* × *b*), where *a* and *b* are the number of classes of ontologies A and B, respectively. Once a window length *W* (default 4) is selected, for each node *i* from A FOntCell constructs the surrounding subgraph of nodes {*i*} ∈ *A*_*w*_(*i*) and calculates its similarity with all subgraphs {*j*}∈ *B*_*w*_(*j*), where *A*_*w*_(*i*) and *B*_w_(*j*) are the subgraphs of length *W* centered in *i* and *j*, respectively. Each subgraph *A*_*w*_(*i*) is defined by a center node *i* and all the nodes inside a window length *W* upstream or downstream of *i*. Thus, FOntCell performs a structure convolutional matching, tailoring different metrics to calculate the similarity between subgraphs *A*_*w*_(*i*) and *B*_w_(*j*).

**Figure 3:**
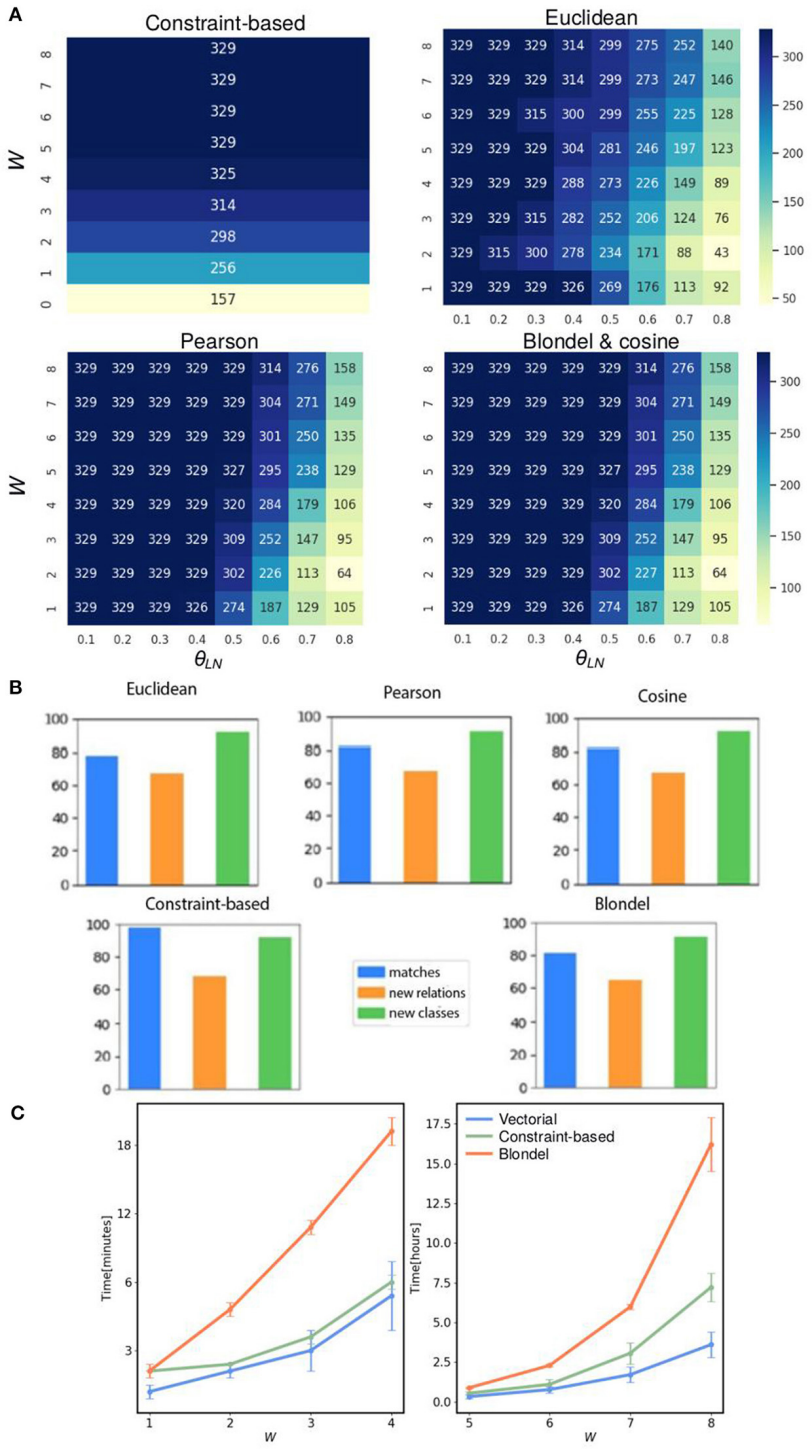
FOntCell performance merging CELDA with LifeMap. **(A)** Heat maps of the matches obtained with two-parameter combinations, window length and name score threshold, using five structure matching methods: the three vectorial structure matching {cosine, Euclidean, Pearson}; constraint-based structure matching, and Blondel structure matching. The two optimized parameters are the window length *W* and the local sequence threshold θ_*LN*_ in the ranges [0.1, 0.8] and [1, 8], respectively, using steps of 0.1 for θ_*LN*_, and 1 for *W*. Bluer color corresponds to higher number of synonyms. **(B)** Percentages of matches, new classes and new relations, obtained with the five structure matching methods with merging alignment parameters *W* = 4, θ_*N*_ = 0.85, and θ_*LN*_ = 0.7. **(C)** Run time for the five structure-matching methods for θ_*N*_ = 0.85, and θ_*LN*_ = 0.7, and window sizes *W* in the range [1, 8]. The vectorial structure matching {cosine, Euclidean, Pearson} have similar run time lines and are represented by a single line.

The Blondel method (Blondel et al., 2004), initially developed to measure the similarity between graph vertices can be used to assess the structure matching between two networks, however it is quite computationally demanding (Figure 3C). To improve the speed of the structure mapping, we designed two new methods that calculate the structure matching of ontologies in a convolutional fashion: Vectorial structure matching and Constraint-based structure matching; additionally, we adapted the Blondel method to work for such new convolutional structure matching approach. An example of a convolution window sliding across a graph is depicted in Figure 2B.

FOntCell takes as generator nodes those without name assignment during the calculation of the name mapping matrix *S*^AB^. The subgraphs generated from these nodes from both ontologies are evaluated using one of the metrics explained below.

### Vectorial Similarity Based on Graph Convolution as a Structure Matching Metric

For each possible pair of nodes *i* ∈ A and *j* ∈ B, and window length *W*, FOntCell extracts the subgraphs *A*_*W*_(*i*) and *B*_*W*_(*j*) of length *W* centered in *i* and *j*, and adjacency matrices ${\tilde{A}}_{W}\left(j\right)$ and ${\tilde{B}}_{W}\left(j\right)$ with number of nodes *a*_*Wi*_ and *b*_*Wj*_, respectively. For all possible pairs of nodes *k* ∈ *A*_*W*_(*i*) and *l* ∈ *B*_*W*_(*j*), FOntCell takes the corresponding rows $\tilde{ik}$ and $\tilde{jl}$ of the adjacency matrices ${\tilde{A}}_{W}\left(i\right)$ and ${\tilde{B}}_{W}\left(j\right)$, and calculates their similarity using one of the *M* = {1 − cosine, Euclidean, 1 − Pearson} metrics. Since the lengths *a*_*Wi*_ and *b*_*Wj*_ of those rows are not necessarily equal, FOntCell calculates all *nc* possible convolution similarities, $p_{ik,jl}^{c}$ of the shorter row over the longer row, where *nc* = abs(a_*Wj*_ *- b*_*Wi*_) + 1 is the number of convolutions and *c* ∈[1, *nc*] (Figure 2B), and selects the maximum similarity: $p_{ik,jl}^{Max}=\max p_{ik,jl}^{c}$. Then, for each (*i,j*) pair, it assigns to the (*i,j*) position of the structure mapping matrix *T*^*AB*^(*i,j*) the maximum similarity $p_{ik}^{Max}=\max\;p_{ik,jl}^{Max}$ for *jl* across ${\tilde{B}}_{W}\left(j\right)$. For brevity, throughout the whole text, we name the vectorial structure matching using the (1 − cosine) and (1 − Pearson) metrics as cosine and Pearson structure matching, respectively.

### Constraint-Based Similarity as a Structure Matching Metric

We developed the constraint-based structure matching based on three assumptions: (i) The matches obtained from the name matching are correct. (ii) The degree of structural similarity of two generator nodes is proportional to the number of matches between the subgraphs generated by them. (iii) Two generator nodes are more likely to be equivalent if their close relatives have name matches.

To calculate the similarity between two generator nodes, *i* and *j*, FOntCell first obtains the number of name matches between the two subgraphs {*i*} and {*j*}. Next, it weighs them according to the proximity to the generator nodes *i* or *j*. The matches closer to the generator node score higher. To implement the method, for all possible pairs of nodes *i* ∈ A and *j* ∈ B, and for a window length *W*, FOntCell searches for all possible name matched pairs between the lists of nodes {*k*} ∈ *A*_*w*_(*i*) and the list {*l*} ∈ *B*_*w*_(*j*), where *A*_*w*_(*i*) and *B*_*w*_(*j*) are the subgraphs of length *W* centered in the generator nodes *i* and *j*, respectively, with the condition that at least one node of the list {*k*} ∈ *A*_*w*_(*i*) has a name match with a node of the list {*l*} ∈ *B*_*w*_(*j*). Then, for each node *k* in the list {*k*}, FOntCell calculates the shortest path *s*_*ki*_ to *i*, and produces a list {*s*_*ki*_} of shortest paths to estimate the proximities to the generator node *i*. FOntCell assigns to each shortest path *s*_*ki*_ a constraint *c*_*ki*_ = *W*+1−*S*_*ki*_. Finally, it sums the list of constraints {*c*_*ki*_} to produce an accumulated constraint *C*_*i*_ and assigns it to the structure mapping matrix *T*^AB^(*i,j*).

### Blondel Similarity as a Structure Matching Metric

To perform graph structure matching FOntCell adapts the original Blondel metric:

(3) $$\begin{array}{l} T_{k+1}^{AB}=\frac{{\tilde{B}T}_{k}^{AB}{\tilde{A}}^{t}+{{\tilde{B}}^{t}T}_{k}^{AB}\tilde{A}}{\left||\tilde{B}T_{k}^{AB}{\tilde{A}}^{t}+{\tilde{B}}^{t}T_{k}^{AB}\tilde{A}|\right|}, \end{array}$$

where *t* is the transpose operator. Equation (3) is calculated iteratively until an even number of steps *k* of convergence to a stable structure matching *T*^AB^ is reached. As with the other metrics, FOntCell takes sets of subgraphs generated from each generator node and calculates the similarity between these nodes using the Blondel metric. For each node *i* from *A*, FOntCell constructs the surrounding subgraph {*i*}∈A and calculates its similarity with all subgraphs {*j*}∈*B* using Equation (3) with the adjacency matrices of each subgraph. FOntCell performs a structure convolution, tailoring Equation (3) to the case of subgraphs {*i*} and {*j*}.

(4) $$\begin{array}{l} T_{k+1}^{\left\{i\right\}\left\{j\right\}}=\frac{\tilde{\left\{j\right\}}T_{k}^{\left\{i\right\}\left\{j\right\}}{\tilde{\left\{i\right\}}}^{t}+{\tilde{\left\{j\right\}}}^{t}T_{k}^{\left\{i\right\}\left\{j\right\}}\tilde{\left\{i\right\}}}{\left||\tilde{\left\{j\right\}}T_{k}^{\left\{i\right\}\left\{j\right\}}{\tilde{\left\{i\right\}}}^{t}+{\tilde{\left\{j\right\}}}^{t}T_{k}^{\left\{i\right\}\left\{j\right\}}\tilde{\left\{i\right\}}|\right|} \end{array}$$

where $\tilde{\left\{i\right\}}$ and $\tilde{\left\{j\right\}}$ are the adjacency matrices of the respective subgraphs {*i*} and {*j*}. Finally, the structure score on the position of *i* and *j* in the $T_{k+1}^{\left\{i\right\}\left\{j\right\}}$ matrix is assigned to *T*^AB^(*i,j*).

Each of the above defined structure matching methods returns a structure score between two nodes (*i,j*) defined by *T*^AB^(*i,j*) where *i* ∈ A and *j* ∈ B. This convolution improves the result of the structure mapping over the whole graphs since it reduces the influence of distant nodes and edges. Name mapping and structure mapping carry complementary information and FOntCell regains information from both.

### Ontology Alignment

To match classes, FOntCell initially selects the best match for each node *i* from ontology A with a node *j* from ontology B, using the name mapping matrix *S*^*AB*^. If *S*^*AB*^(*i,j*) > θ_*N*_, FOntCell considers classes *i* and *j* as matched and classifies this assignment as a ‘name match'. If *S*^AB^(*i,j*) ≤ θ_*N*_, FOntCell takes the element *T*^AB^(*i,j*) from one of the aforementioned structure matching methods selected by the user to calculate the structure mapping and considers the nodes *i* and *j* matched if *T*^AB^(*i,j*) ≥ θ_*T*_, where θ_*T*_ is a structure mapping threshold selected by the user.

### Ontology Local Name Matching

To improve the result achieved with the structure matching method, FOntCell performs a further local name comparison using the name mapping matrix *S*^*AB*^ to calculate the mean of the name match *S*^{i}^^{*j*}^ of each subgraph pair {*i*} {*j*}, with the same window size *W* used to calculate *T*^{i}^^{*j*}^. FOntCell takes the best name scores from *S*^AB^(*i,j*), calculates the mean of these name matching scores for the pair {*i*} {*j*}, and then builds a new name matching matrix of {*i*}{*j*}: *S*^{i}^^{*j*}^. If *S*^{i}^^{*j*}^ > θ_*LN*_, where θ_*LN*_ is a local name matching threshold (default value θ_*LN*_ = 0.7), FOntCell considers nodes *i* and *j* as synonyms and classifies the corresponding classes as a structure match (Figure 1B). FOntCell creates a file with the relevant information about each node from A with five columns: (1st) native node label in A, (2nd) translated node label assigned from B, (3rd) name score, (4th) structure score, and (5th) type of assignment (Name/Structure). In case of no assignment, the type of assignment is marked as Non-matched.

### Ontology Merging

Once the matched classes between two ontologies are detected, FOntCell translates the name/labels of all classes from ontology B to their equivalent names, if any, in ontology A. Next, FOntCell appends the translated classes from B to A and their corresponding offspring relationships. Then, it performs an ordered-set operation to eliminate all the possible class-relations repeats generated from the appendage. The resulting relation array represents the merging of the two ontologies. In addition, FOntCell creates an OWL format file with the result of the merging by reading the .owl file of ontology A and appending the new classes from B at the start of the ontology class site. The information of these new classes is stored in four columns: (1st) new ID, (2nd) class label, (3rd) class synonyms, and (4th) ascendant relationship. Finally, FOntCell creates an .html file with an interactive circular Directed Acyclic Graph (DAG) of the original and merged ontologies, and statistical information of the merging, i.e., percentage and number of added classes/relations and type of matches in textual and graphical form.

### Alignment Performance Scores

To evaluate the performance of FOntCell during alignment and to compare it with other alignment methods, we used the Precision (Equation 5), Recall (Equation 6) and Accuracy (Equation 7) in terms of Type I and Type II errors:

(5) $$\begin{array}{l} \text{Precision}=\frac{\text{TP}}{\text{TP}+\text{FP}} \end{array}$$

(6) $$\begin{array}{l} \text{Recall}=\frac{\text{TP}}{\text{TP}+\text{FN}} \end{array}$$

(7) $$\begin{array}{l} F_{\beta }=\frac{\left(1+{\text{β}}^{2}\right)\cdot \mathrm{Precision}\cdot \mathrm{Recall}}{\left({\text{β}}^{2}\cdot \mathrm{Precision}\right)+\mathrm{Recall}}\\ \;=\frac{\left(1+{\text{β}}^{2}\right)\cdot \mathrm{TP}}{\left(1+{\text{β}}^{2}\right)\cdot \mathrm{TP}+{\text{β}}^{2}\cdot \mathrm{FN}+\mathrm{FP}} \end{array}$$

where β is real positive number that accounts for how many times the precision is considered more important than the recall in the measurement of the accuracy, and TP, FP and FN are the numbers of True Positives, False Positives and False Negatives, respectively. We calculated three accuracies: F_1_, harmonic mean of the precision and the recall; F_0.5_ which gives double weight to the precision compared to the recall, attenuating the false negative influence; F_2_, which gives double weight to the recall compared to the precision, giving more emphasis on false negatives.

To assess the alignment performance for the cases of CELDA+LifeMap and CELDA+LifeMap+ LMHA which are *de novo* alignments without reference ones, we built manually the references and used them to compare the performance of all the alignment tools.

## Results

### Cosine Is the Best Structure Method for the CELDA + LifeMap Merging

To find the optimal parameters of FOntCell for the merging of CELDA with LifeMap, we performed a bidimensional scanning of the alignment parameters: local name threshold θ_*LN*_ and window length, *W*, in the range [0.1, 0.8] and [1, 8], respectively, using steps of 0.1 for θ_*LN*_, and 1 for *W* for all structure mapping metrics: the three vectorial structure matching methods (Euclidean, Pearson, and cosine), the constraint-based structure matching, and the Blondel structure matching (Figure 3A). The constraint-based method does not involve local name matching, therefore θ_*LN*_ was not used. A name mapping threshold θ_*N*_ = 0.85 produces an accuracy F_1_ > 0.9 for almost all the metrics (Supplementary Table 1), thus, we keep θ_*N*_ = 0.85 for the rest of the analysis. θ_*N*_ > θ_*LN*_ recovers some meaningful cases during the structure mapping and helps to overcome the graph isomorphism problem arising during subgraph comparisons. The name mapping threshold θ_*N*_ = 0.85 assigns as similar class labels those labels that differ in orthographic variations, such as “s” endings, apostrophes, etc. Therefore, we set for the remaining analysis θ_*N*_ > θ_*LN*_ = 0.7 since we expected more name variability in nodes between subgraphs comparisons than in class-to-class comparison. It is important to reduce the θ_*LN*_ sensitivity since a more sensitive method finds more isomorph subgraphs. Smaller *W* produces smaller subgraphs, increasing the possibility to slip into isomorph subgraphs making the structure metric more sensitive to θ_*LN*_, while for very large *W*, FOntCell merges unrelated subgraphs of the two ontologies. For the CELDA and LifeMap merging, the window size that minimizes the sensitivity to θ_*LN*_ is *W* = 4. The constraint-based method with *W* = 4 slips into subgraph isomorphism, i.e., it finds too many synonyms and has higher sensitivity to the change of *W* than other vectorial methods (Figure 3A). The Euclidean method is more restrictive than the other vectorial methods but more sensitive to θ_*LN*_ (Figure 3A). The Pearson and cosine methods produce almost the same number of matches for all combinations of alignment parameters (Figure 3A). The cosine method obtains exactly the same number of synonyms as the Blondel method for all pairs of parameters (Figure 3A).

To analyze the effect of each of the five structure mapping methods on the percentages of matches, new classes and new relations between them, and to find the best structure mapping method, we performed a FOntCell merging of CELDA and LifeMap for the optimized alignment parameters: *W* = 4, θ_*N*_ = 0.85 and θ_*LN*_ = 0.7, for each structure matching method. We found similar number of classes and relations added by the different structure matching methods, and similar number of matches (Figure 3B).

We studied the run time of the five structure mapping methods for the optimized alignment parameters θ_*N*_ = 0.85 and θ_*LN*_= 0.7, and window sizes *W* in the range [1, 8], and we found that the vectorial methods (cosine, Euclidean and Pearson) are the fastest, and at least one order of magnitude faster that the Blondel method (Figure 3C). Since the vectorial methods are much faster than the Blondel method, and among them the cosine method obtains the same number of synonyms as the Blondel, we chose to use the cosine method in the remaining analysis.

### The Merging of CELDA With LifeMap Expanded CELDA by 67%

**Figure 4:**
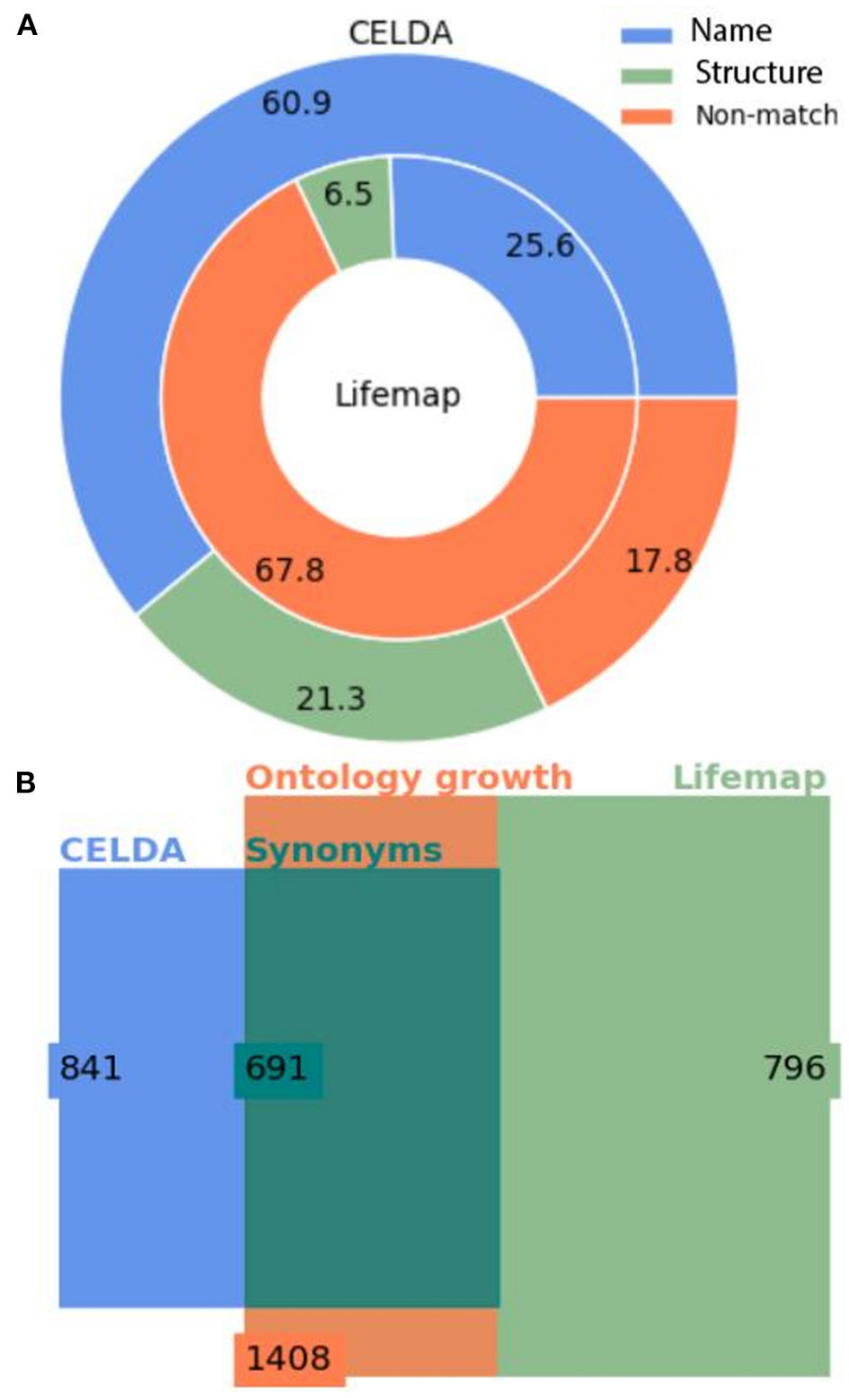
Statistics of the merging of CELDA and LifeMap with the cosine structure matching metric. **(A)** Donut plot of the percentages of classes added by name mapping vs. the classes added by structure mapping to CELDA (outer circle) from LifeMap (inner circle). **(B)** Square Euler-Venn diagram with the number of classes before and after merging. The blue and light green rectangles frame the number of classes in CELDA and LifeMap, respectively, before the merging, the dark green rectangle frames the sum of name and structure equivalent classes, and the orange rectangle frames the total number of classes in the resultant CELDA and LifeMap merged ontology. Alignment parameters *W* = 4, θ_*LN*_ = 0.7 and θ_*N*_ = 0.85.

**Figure 5:**
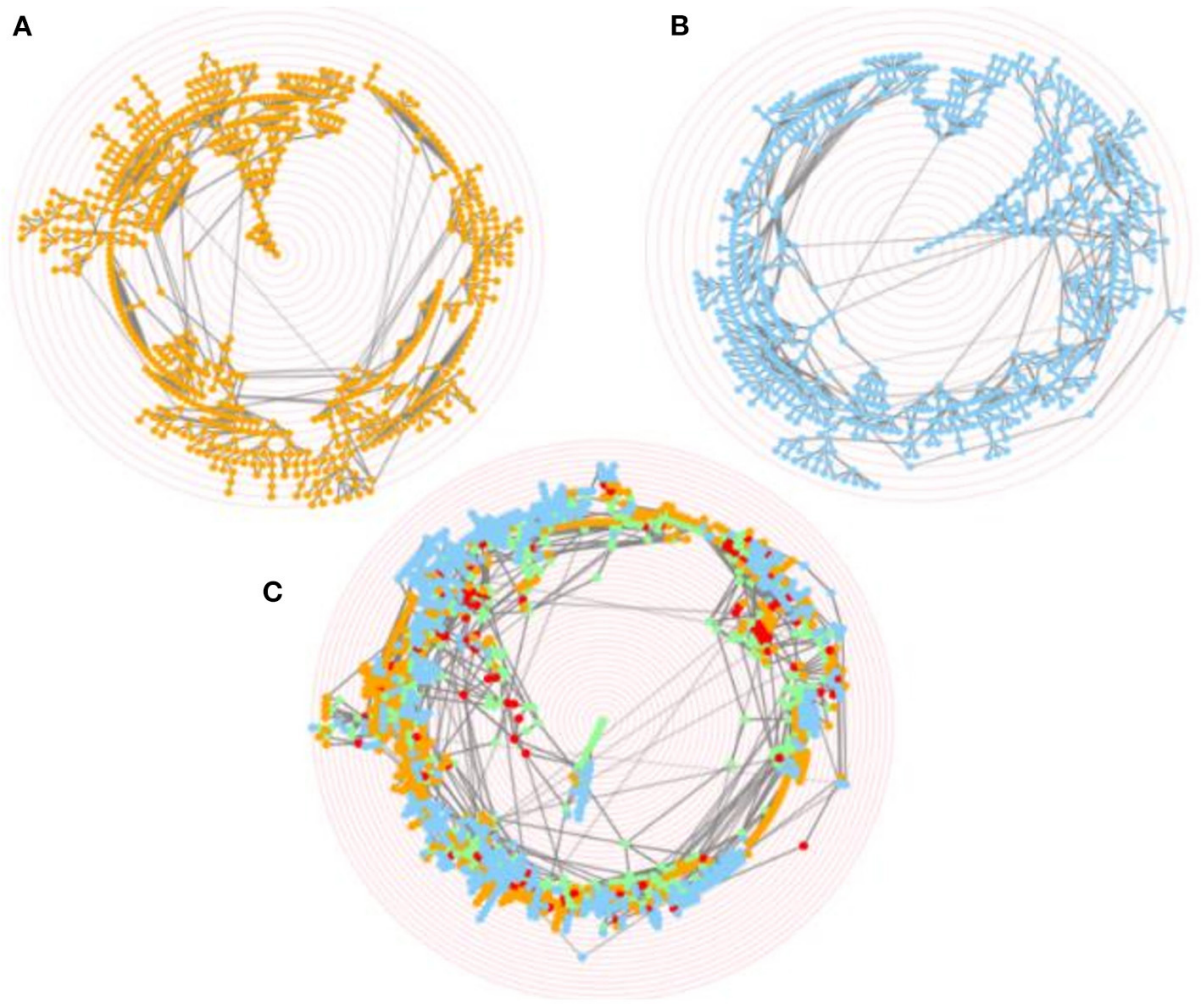
Merging of CELDA and LifeMap ontologies. Screenshots of the interactive circular Directed Acyclic Graphs (DAGs) of **(A)** CELDA, **(B)** LifeMap and **(C)** the merged CELDA + LifeMap ontology, respectively. The orange and blue nodes are the non-matched contributions from ontology A and ontology B, respectively. The green and red nodes are the nodes with name and structure mapping, respectively. The ontology labels associated to the nodes appear when hovering over the nodes. The concentric red rings are zoom guides.

**Figure 6:**
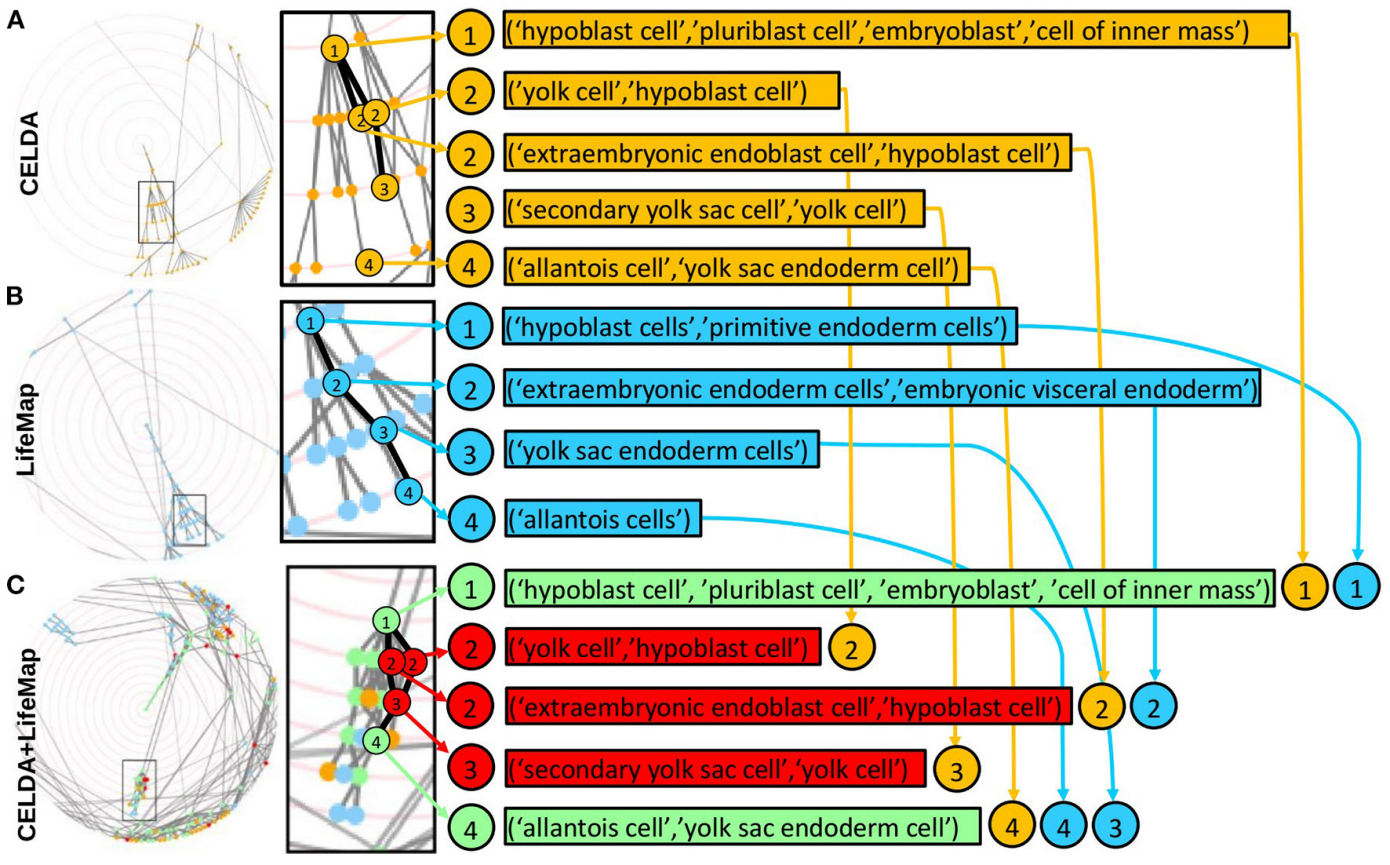
Zooms of regions of CELDA, LifeMap and the merged ontology where FOntCell performs name and structure mapping. (Left) Screenshots of the interactive circular Directed Acyclic Graphs (DAGs) of **(A)** CELDA, **(B)** LifeMap and **(C)** the merged CELDA+LifeMap ontology. (Right) Zoomed regions with corresponding lists of cell types. The synonymous names in each list are separated by commas. The orange and blue nodes are the non-matched contributions from CELDA and LifeMap, respectively. The green and red nodes are the nodes with name and structure mapping, respectively. The numbers inside circles indicate the relative parent-child relationship in ascending order.

The merging of CELDA and LifeMap with θ_*LN*_ = 0.7 and *W* = 4 resulted in an ontology integrating all the 841 classes from CELDA with 567 classes from LifeMap. Thus, the merged ontology increased the cell ontology information of CELDA by 67% (Figure 4A) with accuracy F_1_ = 0.9 (Supplementary Table 1). The generated by FOntCell interactive DAGs of CELDA, LifeMap and the resultant merged ontology are presented in Figure 5. Zooms of regions where FOntCell performed both name and structure mapping (Figure 6) illustrates some of the challenges arising during alignment of cell ontologies, and how the structural matching rescues information from one ontology to augment the other ontology and enhanced the final merged ontology: Two or more classes of CELDA can align with one class of LifeMap, a rather common phenomenon when activating the use of synonyms. In the zoomed regions CELDA (Figure 6A) and LifeMap (Figure 6B) have similar but not identical structures. CELDA starts with “hypoblast cell,” with children “yolk cell” and “extraembryonic endoblast cell,” with the latter further having as a child “secondary yolk sac”, whereas in LifeMap the same developmental region starts with “hypoblast cell,” followed by “extraembryonic endoderm cells,” “yolk sac endoderm cells”, and finally “allantois cell.” The merging shows a consensus (Figure 6C) that starts with “hypoblast cell” (as in CELDA and LifeMap), that as child cell has “yolk cell” from (CELDA and LifeMap) and incorporates as an additional child the “extraembryonic endoblast cell” owing to the information provided by LifeMap. From these two children, resulting from the merging of similar but not identical cells follows the “secondary yolk” that additionally incorporates as a child the “allantois cell” owing to the structural information provided by LifeMap.

For a less restrictive pair of parameters θ_*LN*_ = 0.1 and *W* = 1 using the cosine metric, 39.1% of classes from CELDA have a structure matching in LifeMap independently of the used structure matching method. For more restrictive parameters θ_*LN*_ = 0.7 and *W* = 7, we obtained 32.2% of classes with structure mapping.

### Aligning CELDA and LifeMap, FOntCell Has Precision of 99% With Name Mapping, and Mean Precision of 55% With the Five Structure Mapping Methods

**Figure 7:**
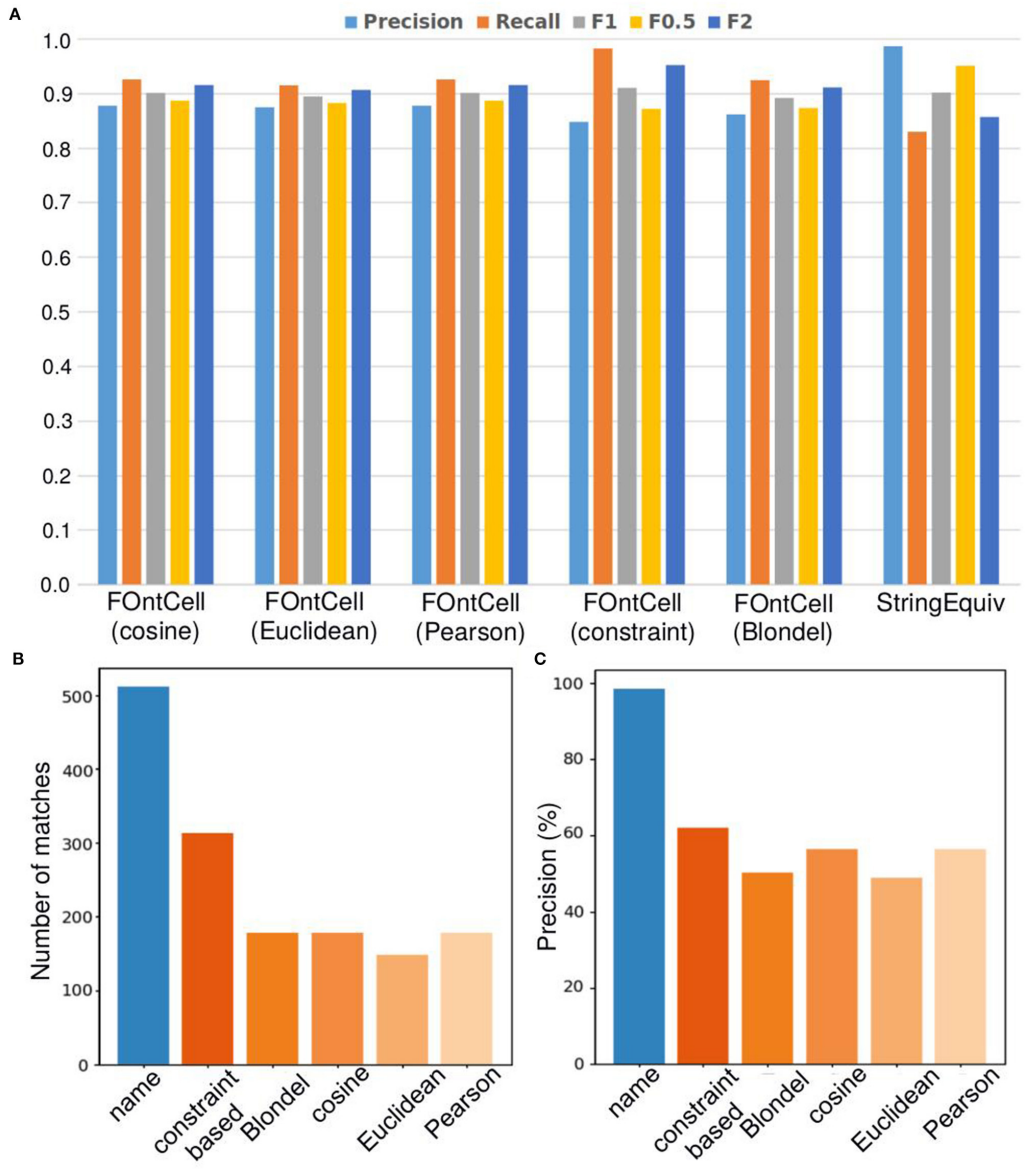
Alignment performance of the different mapping methods of FOntCell when merging CELDA and LifeMap. **(A)** Precision, recall and F_β_ alignment accuracies of the different structure mapping methods combined with name mapping (FOntCell), and of name mapping applied separately (StringEquiv). **(B)** Number of matches during ontology matching with the different mapping methods. Name mapping is shown in blue and the structure mappings in different hues of orange. **(C)** Precision of the name matching and the different structure mapping methods. Name mapping is shown in blue and the structure mappings in different hues of orange. Alignment parameters *W* = 4, θ_*LN*_ = 0.7 and θ_*N*_ = 0.85.

We calculated the precision of the different mapping methods of FOntCell when merging CELDA and LifeMap with the optimal parameters *W* = 4, θ_*LN*_ = 0.7 and θ_*N*_ = 0.85 (Figure 7A). The results obtained through name mapping and the different structure mapping methods were validated checking failures, false positives (FP) and successes, true positives (TP) on the matching of the cell types and calculating the precision using Equation (5). The name matching shows 98.63% precision (Figure 7A) and has the highest number of matches (Figure 7B), 512, in comparison with the other matching methods of FOntCell. Among the structure mapping methods, highest precision of 62.10% is shown by the constraint-based method, followed by the cosine and the Pearson, 56.42%, Blondel, 50.27%, and the Euclidean, 48,99%, methods (Figure 7C). Evaluating the whole FOntCell mapping process, name and structure mapping methods taken together, we observe similar total precision with all the methods: ~87% using the vectorial methods, 86.1% with the Blondel method, and 86% with the constraint-based method. Anyway, the vectorial methods produce higher total precision values due to the contribution of fewer matches than in the constraint-based and in the Blondel methods.

When considering only structure mapping precision, the Blondel method is the second worst one, slightly better than the Euclidean method (Figure 7C). However, when combined with name mapping, all the vectorial methods, including the Euclidean method, surpass the precision of the Blondel method (Figure 7A) due to the Blondel method producing more matches during the structure matching than the Euclidean (Figure 7B). This indicates that the synergies arising between name mapping and structure method combinations are stronger for the vectorial methods than for the Blondel method, at least in the case of CELDA and LifeMap merging.

Considering only the structure mapping precision, the Euclidean method has the lowest one, 48.99% (Figure 7C), while combined with name mapping it has precision of 87.44%, similar to the combined precision of the other vectorial methods, cosine and Pearson (Figure 7C), because of the low number of matches obtained during structure matching, which is actually the lowest (Figure 7B). This indicates that the synergies arising between name mapping and structure method combinations equilibrate for all the vectorial methods. The constraint-based method contributes the highest number of matches (Figure 7B), and although it has the highest precision of 62.1% among the structure mapping methods (Figure 7C), it has the lowest total precision among the combined methods (Figure 7A).

The Pearson and cosine methods show equal performance, both with the same number of matches, 179 (Figure 7B), and the same structure mapping precision of 56.42% (Figure 7C), which results a total precision of 87.69% when combined with the name mapping, a total precision of 87.69% when combined with the name mapping (Figure 7A). In conclusion, the cosine and Pearson methods in combination with name matching achieve the highest total precision and the smallest number of matches. Therefore, we chose the cosine method as default structure matching method of FOntCell. Anyway, we could have chosen the Pearson structure method with equally good results.

### The Merging of CELDA and LifeMap Has an Accuracy F_1_ of 0.91

We calculated the precision (Equation 5), the recall (Equation 6) and the family of accuracies F_β_: F_1_, F_0.5_ and F_2_ (Equation 7) with the optimal parameters: *W* = 4, θ_*LN*_ = 0.7 and θ_*N*_ = 0.85, for each of the structure metrics (cosine, Euclidean, Pearson, constraint-based, Blondel), and an additional metric that only measures the string similarity.

All metrics produce CELDA and LifeMap alignments with similar F_β_ accuracies. The Pearson and cosine obtain slightly higher F_1_ and F_2_ accuracies than the Euclidean for F_1_ and F_2_ due to the Euclidean slightly lower recall. The Blondel method exhibits similar to the vectorial methods behavior with a slight decrease in precision and a recall similar to cosine and Pearson. The constraint-based method has lower precision but higher recall (Supplementary Table 1).

For all FOntCell alignment methods, the precision ranges between 0.847 and 0.877, and the recall between 0.982 and 0.915 (Supplementary Table 1). In the name mapping case (StringEquiv), where structural alignment is not used, the precision is close to 1 but the recall nevertheless decreases considerably (Supplementary Table 1); thus the name mapping misses to align numerous classes and many of them are aligned by the structure alignment methods.

After merging the aligned the ontologies, the resulting ontologies with the cosine and Pearson methods have a greater number of matches and a greater growth in the number of classes (Figure 3C) compared to the Euclidean method. The parameters that influence more the process in terms of increasing the number of classes and the number of matches in the final ontology are *W* and θ_*LN*_. For all values of θ_*N*_, the number of structure matches found have an inflection point when *W* = 4 and θ_*LN*_ = 0.7 (Figure 3A). This is the midpoint where the method is not restrictive enough and not excessively permissive.

The CELDA + LifeMap merging with the optimal configuration parameters: *W* = 4, θ_*LN*_ = 0.7, and the cosine vector method found 691 synonyms between the two ontologies and generated a final cell ontology with 1,408 classes, 841 from CELDA and 1,408 – 841 = 567 added classes from LifeMap (Figure 4B).

### The F_β_ Alignment Accuracies of FOntCell Are Above the Geometric Mean When Comparing With Other Alignment Tools of the OAEI

To compare the alignment capability of FOntCell with other tools in a different problem, we selected the alignment of mouse and human anatomy ontologies task proposed by the Ontology Alignment Evaluation Initiative (OAEI) in 2019. We used the optimized parameters: *W* = 4, θ_*LN*_ = 0.7 and θ_*N*_ = 0.85, and performed the analysis for all the structural metrics implemented in FOntCell. For all metrics and the whole family of accuracies F_β_, FOntCell performs above the geometric mean of the other tools (Table 3). The simple name matching, incorporated in FOntCell as a complementary alignment, is more precise but with lower recall, leading to lower F_1_ and F_2_ but higher F_0.5_ accuracies. The different types of structural alignment of FOntCell find new classes undiscovered by the name mapping alignment.

**Table 3:** Performance scores of FOntCell and other tools in the alignment of the anatomy ontologies of OAEI 2019.

	**Precision**	**Recall**	**F_**1**_**	**F_**0.5**_**	**F_**2**_**
FOntCell (cosine)	0.861	0.720	0.784	0.829	0.744
FOntCell (Euclidean)	0.909	0.726	0.807	0.865	0.756
FOntCell (Pearson)	0.859	0.724	0.786	0.828	0.748
FOntCell (constraint)	0.846	0.718	0.777	0.817	0.740
FOntCell (Blondel)	0.860	0.724	0.786	0.829	0.748
StringEquiv	0.997	0.622	0.766	0.890	0.673
AML	0.950	0.936	0.943	0.947	0.939
LogMap	0.918	0.846	0.881	0.903	0.859
AGM	0.152	0.195	0.171	0.159	0.185
ALIN	0.974	0.698	0.813	0.903	0.740
DOME	0.996	0.615	0.760	0.886	0.666
FCAMap-KG	0.996	0.631	0.773	0.893	0.681
Lily	0.873	0.796	0.833	0.856	0.810
LogMapBio	0.872	0.925	0.898	0.882	0.914
LogMapLite	0.962	0.728	0.829	0.904	0.765
POMAP++	0.919	0.877	0.898	0.910	0.885
SANOM	0.888	0.844	0.865	0.879	0.852
GeoMean	**0.807**	**0.683**	**0.735**	**0.775**	**0.702**

*In the case of FOntCell, the name of the used structure mapping method is inside parentheses. GeoMean are the geometric mean values of the performance scores of the other tools with which FOntCell is compared*.

### FOntCell Outperforms Significantly the Best OAEI Tools in the CELDA and LifeMap Alignment in Terms of F_β_ Alignment Accuracies

We selected the best performing tools that we found during the alignment of mouse and human anatomy ontologies (Table 3): StringEquiv, AML and LogMap, and ran them with their default parameters to compare their performance with FOntCell in the case of the alignment of CELDA and LifeMap. They showed higher precision but a lower recall than FOntCell, especially in the case of AML and LogMap. The lower recall penalized their accuracy leading to lower F values (Table 4).

**Table 4:** Performance scores of FOntCell and other tools in the alignment of CELDA and LifeMap.

	**Precision**	**Recall**	**F_**1**_**	**F_**0.5**_**	**F_**2**_**
FOntCell (cosine)	0.877	0.925	0.900	0.886	0.915
FOntCell (Euclidean)	0.874	0.915	0.894	0.882	0.906
FOntCell (Pearson)	0.877	0.925	0.900	0.886	0.915
FOntCell (constraint)	0.847	0.982	0.910	0.871	0.952
FOntCell (Blondel)	0.861	0.924	0.891	0.873	0.911
StringEquiv	0.986	0.829	0.901	0.950	0.857
AML	0.971	0.269	0.422	0.639	0.315
LogMap	0.983	0.317	0.480	0.692	0.367
GeoMean	**0.980**	**0.413**	**0.567**	**0.749**	**0.463**

*In the case of FOntCell, the name of the used structure mapping method is inside parentheses. GeoMean are the geometric mean values of the performance scores of the other tools with which FOntCell is compared*.

The F_1_ accuracies of StringEquiv and the different methods of FOntCell alignments are similar, ~0.9. For the accuracy that gives more weight to the precision, F_0.5_, StringEquiv outperformed the other methods. For the accuracy that gives more weight to the recall, F_2_, FOntCell that combines a name matching with a structure matching obtained a better result. Importantly, when merging is oriented at complementing two ontologies, the recall is a key value to be able to rescue many new cell types, and all metrics of FOntCell outperform the other methods in this case. Noteworthy, for the whole family of accuracies F_β_, all FOntCell metrics outperform significantly the best OAEI tools in the CELDA and LifeMap alignment (Table 4).

### The Merging of CELDA + LifeMap With LMHA Generates 65 New Relations and 39 New Classes

**Figure 8:**
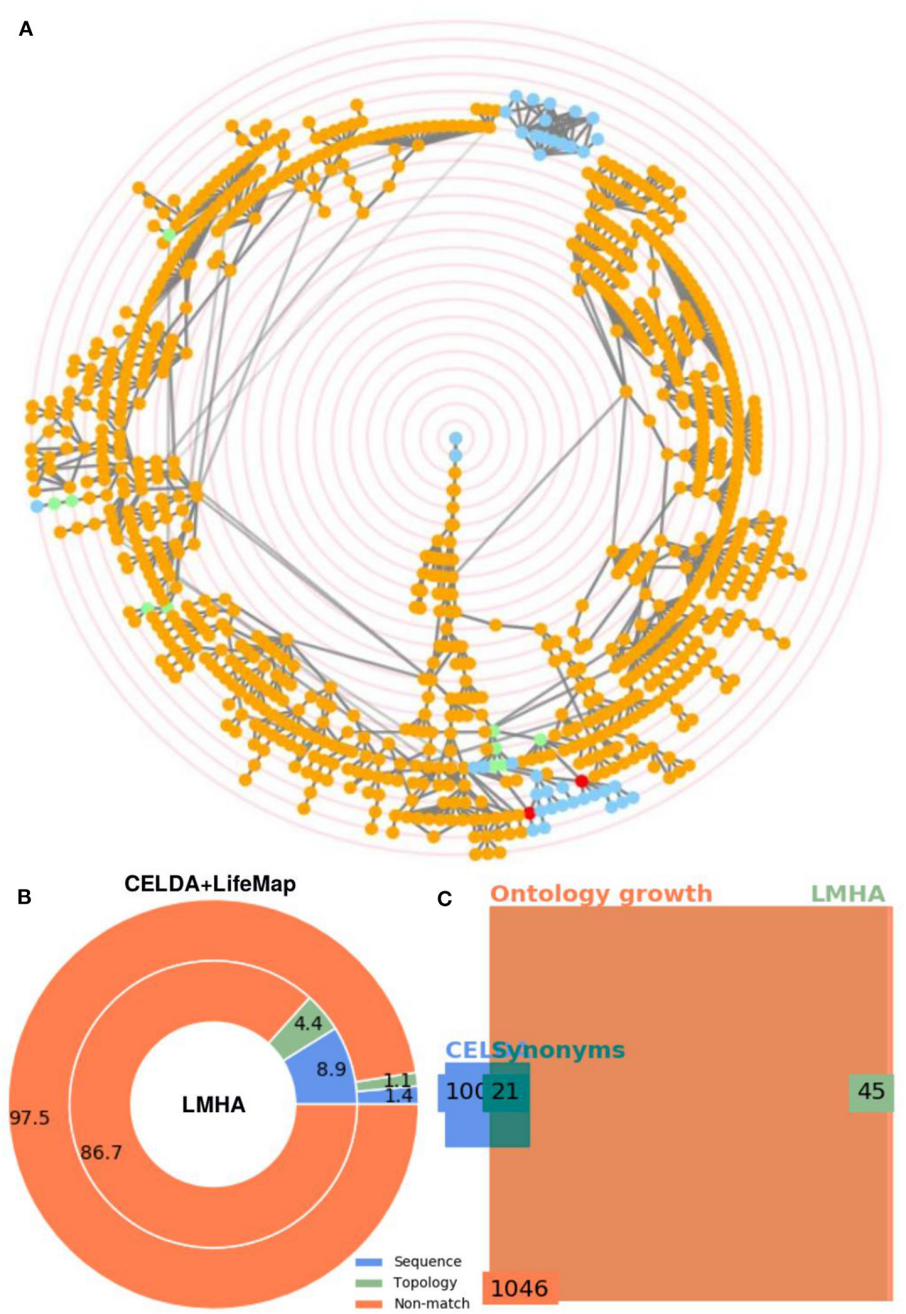
Merging of CELDA + LifeMap with LungMAP Human Anatomy (LMHA) ontology. **(A)** Circular Directed Acyclic Graph (DAG) of the merged ontology. The orange and blue nodes are the non-matched contributions from CELDA+LifeMap and LMHA, respectively. The green and red nodes are the nodes with name and structure mapping, respectively. In the interactive application generated automatically in html by FOntCell, the ontology labels associated to the nodes appear when hovering over the nodes. The concentric red rings are zoom guides. **(B)** Donut plot of the percentages of classes added by name mapping vs. the classes added by structure mapping to the merged CELDA + LifeMap (outer circle) from LMHA (inner circle). **(C)** Square Euler-Venn diagram with the number of classes before and after the merge. The blue and light green rectangles frame the number of classes in CELDA + LifeMap and LMHA before the merging, respectively, the dark green rectangle frames the sum of name and structure equivalent classes, and the orange rectangle frames the total number of classes in the resultant CELDA + LifeMap + LMHA merged ontology. Alignment parameters *W* = 4, θ_*LN*_ = 0.7 and θ_*N*_ = 0.85.

One of the applications of FOntCell is to merge an ontology from a broad, general description, with another ontology with very specific knowledge within the same knowledge domain. In order to illustrate this functionality, we merged the ontology resulting from CELDA + LifeMap merging with LMHA, a specific ontology of cells for lung development starting ~36 weeks of human fetal gestation and continuing after birth with some variation in when the alveolar stage commences and when it is complete. The .owl file used in the merging was generated by Susan E Wert, Gail H. Deutsch, Helen Pan, and the National Heart, Lung and Blood Institute (NHLBI) Molecular Atlas of Lung Development Program Consortium Ontology Subcommittee (LungMAP) [U01HL122642] and downloaded from (www.lungmap.net) of the LungMAP Data Coordinating Center (1U01HL122638) of the NHLBI, on April 7, 2018. The merging of CELDA + LifeMap and LMHA produced 65 new relations and 39 new classes related to endothelial and lymphoid cells (Figure 8).

## Discussion

The discovery of new cell types such as those produced by the HCA consortium or their better characterization by single cell transcriptomics (Gerovska and Araúzo-Bravo, 2016) can render old cellular development ontologies obsolete. We developed FOntCell to address this problem with a novel algorithm that by merging ontologies adds new relationships and classes to a base ontology. Such algorithm allows us to construct from two ontologies a cell ontology that is as complete and up-to-date as possible. We implemented FOntCell as a new Python module that merges efficiently ontologies in the same or similar knowledge domains. It processes intra- and inter- ontology synonyms. To process intra-synonyms, its name similarity search engine is equipped with a name list processing functionality. To search for inter-ontology synonyms, FOntCell integrates the name similarity search engine with a structural similarity search based on graph convolution. Since the structural similarity assessment is a lengthy process that takes the highest percentage of the running time of the merge process, to perform the graph convolution we designed two methods to perform structural convolution: vectorial topological similarity and constraint-based topological similarity. To calculate the vectorial topological similarities we designed a general method to calculate the similarities between vectors of different lengths for different metrics. Additionally, we adapted the Blondel method to work for such new topological convolution approach.

Different ontologies could benefit from different alignment parameters; e.g., for the CELDA + LifeMap merging, we found the vectorial methods produce similar results, with a slight advantage for the cosine method. All the functionalities of FOntCell allow the unification of dispersed knowledge in one domain into a unique ontology. FOntCell produces the results in commonly used ontology format files that can be re-used by FOntCell in an iterative way to adapt continuously the ontologies with the new data, endlessly produced by data-driven classification methods. To navigate across the merged ontologies, it generates HTML files with interactive circular DAGs.

FOntCell is a targeted tool for merging cell development ontologies. The objective behind this tool is the production of a cell-type ontology, which bases its relationships on development and serves as the basis for other works that require a holistic vision of cell development. FOntCell helps us collect the information within the different cell-type ontologies and contrasts them against each other without requiring standards or supervision, and grants us a final ontology that contains the cell types that are common and those that are not common between the two. FOntCell, being devised with this objective, does not obtain the same results when it tries to merge other types of ontologies which correspond to other internal hierarchies. Then, the performance scores obtained when merging two ontologies from domains other than cell types are above the average with respect to the rest of the OAEI tools. However, for these cases, there are less specific algorithms that are capable of aligning the ontologies, some of them better than FOntCell.

## Implementation and Software Availability

FOntCell is developed in Python v3.7 and uses the Python library NetworkX to derive the digraph relation of the ontology and to transform each class to a node and each hierarchy step to an edge. NetworkX graphs allows FOntCell access the sorted list of nodes without repeats, and produce digraphs compatible with graph visualization tools such as graphviz and matplotlib. For specific data manipulation, FOntCell uses numpy, pyexcell_ods, argparse, stringdist, and basic Python libraries such as os, collections and itertools. As other merging algorithms (Faria et al., 2018) the algorithm complexity (Big O) is quadratic time O(*n*^2^), however it is possible to reduce the time complexity in the matching problem from quadratic to linear implementing a hash-based searching strategy. For parallelization and the structure-mapping, FOntCell uses BigMPI4py (Ascension and Araúzo-Bravo, 2020). We added a demo function to the FOntCell distribution package merging CELDA with LifeMap.

The automatic installation installs all the dependencies. Additional installation information is provided at https://www.arauzolab.org/tools.html and at https://pypi.org/project/fontcell/. Full instructions of the prerequisites for installation, the downloading of FOntCell, the user manual, an example of how to run FOntCell and an example of the html output created by FOntCell are provided in the Supplementary Material.

## Data Availability Statement

The original contributions presented in the study are included in the article/Supplementary Material, further inquiries can be directed to the corresponding author/s.

## Author Contributions

JC-L and MA-B: conceptualization. JC-L: data curation, software, and validation. JC-L, DG, and MA-B: formal analysis, investigation, methodology, writing-original draft preparation, and writing-review and editing. DG and MA-B: funding acquisition and project administration, resources, and supervision. JC-L, AA, MA-E, and MA-B: visualization.

## Conflict of Interest

The authors declare that the research was conducted in the absence of any commercial or financial relationships that could be construed as a potential conflict of interest. The handling editor declared a past collaboration with one of the authors MA-B.
