# Supplementary material for: FOntCell: Fusion of Ontologies of Cells
Source: Front Cell Dev Biol. 2021 Feb 11;9:562908. doi: 10.3389/fcell.2021.562908 (PMC7905052; doi:10.3389/fcell.2021.562908)
Supplement: Supplementary file 1 [file Data_Sheet_1.ZIP › AdditionalRawFiles/FOntCell_SupplementaryMaterial.pdf]

Supplementary material

FOntCell: Fusion of Ontologies of Cells

Javier Cabau-Laporta[1], Alex M. Ascensión[1], Mikel Arrospide-Elgarresta[1], Daniela Gerovska[1,2,*], Marcos J. Araúzo-Bravo[1,2,3,4,5,6,*]

[1]Computational Biology and Systems Biomedicine, Biodonostia Health Research Institute, San Sebastián, 20014 Spain.
[2]Computational Biomedicine Data Analysis Platform, Biodonostia Health Research Institute, San Sebastián, 20014 Spain.
[3]IKERBASQUE, Basque Foundation for Science, Bilbao, 48013 Spain
[4]CIBER of Frailty and Healthy Aging (CIBERfes), Madrid, Spain
[5]TransBioNet Thematic Network of Excellence for Transitional Bioinformatics, Barcelona Supercomputing Center. Spain.
[6]Computational Biology and Bioinformatics, Department Cell and Developmental Biology Max Planck Institute for Molecular Biomedicine, Röntgenstr. 20, 48149 Münster, Germany
*To whom correspondence should be addressed
Contact e-mail: daniela.gerovska@biodonostia.org, mararabra@yahoo.co.uk

FOntCell is a software module in Python for automatic computed fusion of ontologies. **FOntCell** produces the results of the merged ontology in OBO format that can be iteratively reused by FOntCell to adapt continuously the ontologies with the new data, such of the Human Cell Atlas, endlessly produced by data-driven classification methods. To navigate easily across the fused ontologies, it generates HTML files with tabulated and graphic summaries, and an interactive circular Directed Acyclic Graphs of the merged results.

This document contains:

- The prerequisites for installation of **FOntCell**.
- The instructions to download **FOntCell**.
- The User manual of **FOntCell**.
- Example of how to run **FOntCell**.
- Example of the html output created by **FOntCell**.
- Table S1. Accuracies of CELDA and LifeMap alignment with the different functionalities of **FOntCell** and the String Equivalence method.

Prerequisites for installation of FOntCell

Python3

pip3

It is necessary to use pip3 to download **FOntCell** and use it with Python3.
To install pip3 over Python3 use the command:

```
sudo apt-get install python3-pip
```

TKinter

Tkinter library is a dependency that will not be installed during **FOntCell** installation.
If TKinter has not been previously installed, an error will occur during **FOntCell** import.
To install TKinter over Python3 use the command:

```
sudo apt-get install python3-tk
```

mpi.h

Mpi.h is an mpi4py dependency that might not have been installed during mpi4py installation, and if it is had not been installed, an error might occur.
To install mpi.h use the command:

```
sudo apt-get install libopenmpi-dev
```

Instructions to download FOntCell

FOntCell module is available at PyPI and can be installed using the command:

```
sudo pip3 install FOntCell
```

User manual of FOntCell

After downloading and installing **FOntCell** using pip3, the user has to create the following directories:

output_folder

Is the directory where **FOntCell** will output the results:

- Fused ontology file
- html files
- Figures

input_folder

The input_folder should contain a Configuration file, the two ontologies, and the ontology_edit documents, that should be placed there by the user.

Files required in the Input directory

Configuration file

The configuration file should have the following arguments in .txt format.
Most arguments will be for ontology 1 (A) and ontology 2 (B)

Arguments	Type	Description

input_folder	*str*	path to input directory
output_folder	*str*	path to output directory
Parallelization	*bool*	if True, perform parallelization
Proc	*int*	number of processors[1]
parse_ontology1	*bool*	if True, perform parsing of ontology1
parse_ontology2	*bool*	if True, perform parsing of ontology2
file1	*str*	path to ontology1[2,3]
file1	*str*	path to ontology2[2,3]
take_synonyms1	*bool*	if True, take also the synonyms from ontology1 classes[3]
take_synonyms2	*bool*	if True, take also the synonyms from ontology2 classes[3]
ontologyName1	*str*	name of ontology1[3]
ontologyName2	*str*	name of ontology2[3]
synonym_type1	*str*	*one or more*, ontology1 arguments specifying the location of the labels of the synonyms[3]
synonym_type2	*str*	*one or more*, ontology2 arguments specifying the location of the labels of the synonyms[3]
label_type1	*str*	*one or more*, ontology1 arguments specifying the location of the labels of the classes[3]
label_type2	*str*	*one or more*, ontology2 arguments specifying the location of the labels of the classes[3]
relative_type1	*str*	*one or more*, ontology1 arguments specifying the location of the IDs of the ascendants[3]
relative_type2	*str*	*one or more*, ontology2 arguments specifying the location of the IDs of the ascendants[3]
file_clean_ontology1	*str*	name of the script file with the instructions to **FOntCell** for the automatic editing of ontology1[2,3]
file_clean_ontology2	*str*	name of the script file with the instructions to **FOntCell** for the automatic editing of ontology2[2,3]
del_old_trials_files	*bool*	if True, deletes old files. **Recommended for every new use of FOntCell**
onto_fuse_classes1	*bool*	if true, fuse the classes in ontology1 if they have the same label
onto_fuse_classes2	*bool*	if true, fuse the classes in ontology2 if they have the same label
onto_restriction1	*bool*	if true, fuse the classes in ontology1 if they have the same ID (or secondary info) and the same label
onto_restriction2	*bool*	if true, fuse the classes in ontology2 if they have the same ID (or secondary info) and the same label
onto_list_clear1	*str*	*one or more*, delete the introduced words from labels of ontology1[4]
onto_list_clear2	*str*	*one or more*, delete the introduced words from labels of ontology2[4]
Semantical	*bool*	if true, perform the synonyms with label name matching
text_process	*bool*	if true, perform test processing
split_from1	*str*	split and take the labels of ontology1 from the introduced word to end[4]
split_from2	*str*	split and take the labels of ontology2 from the introduced word to end[4]

split_since1	*str*	split and take the labels of ontology1 from the beginning since the word introduced[4]
split_since2	*str*	split and take the labels of ontology2 from the beginning since the word introduced[4]
Windowsize	*int*	size of the convolutional window (in edges)
Namethreshold	*float*	threshold between [0.0-1.0] for name mapping
Localnamethreshold	*float*	threshold between [0.0-1.0] for local name mapping (used in structure mapping)
structure_threshold	*float*	threshold between [0.0-1.0] for structure mapping*
structure_method	*str*	structure matching type: 'blondel', 'cosine', 'euclidean', 'pearson' or 'constraint-based'
Automatic	*bool*	if true run FOntCell automatic (unsupervised), if false run FOntCell semi-automatic, user control at structure alignment

** Argument can be blank if your analysis does not require those arguments*
[1] only if parallelization is True
[2] files must be at input directory
[3] only if parse ontology is True
[4] if textprocess is True

Every argument of the configuration.txt file needs to be precede by a a '>' character forward to be parsed.

The user does not have to introduce a threshold for a test that it has not been selected

Ontology edition files

These files allows the user to edit the ontology after parse in order to direct the ontology fusion.

The document must be in .txt file with the following instructions defining how to automatic edit the ontology file:

Instruction	Syntax	Description	Example
delete class	'...'	deletes the nodes/classes that contains the label	'ontology label x'
concatenate classes	[[...], [...]]	concatenates two classes, allows introduce new classes	[[[ontology_class_synonyms]1, ID:0], [[ontology_class_synonyms2], ID:1]]
delete class from ID	ID: '...'	deletes the class that has the ID introduced	ID:'xxxxx'
fuse classes	f[[...], [...]]	fuses two classes. The resultant class conserves the ID from the first class, and the descendant-ascendant relations)	f[[ontology_synonyms1], [ontology_synonyms2]]

Ontology files

The ontologies to be fused can be in .owl format (that requires a parse) or in a .ods format, if one wishes to save the parse step.

The .ods file has the graph-edge information in two columns as in the following example:

Ascendant class	Descendant class
[[class synonyms 1], ID class 1]	[[class synonyms 2], ID class 2]
[[class synonyms 1], ID class 1]	[[class synonyms 3], ID class 3]
[[class synonyms 2], ID class 2]	[[class synonyms 4], ID class 4]

The first column corresponds to 'parent' and the second column to the 'descendant'. The possible synonyms of each class are separated by commas.

Files created in the Output directory after fusion

.owl file

This document is the resultant ontology from the fusion of two ontologies. The structure is the same as of ontology 1 (A), with the new classes added from ontology 2 (B) at the top of ontology class section.

FOntCell_OntologyA_OntologyB.html file

This file contains information about the fusion. First, one can see three interactive circular graphs: ontology A, ontology B and the merged ontology. Additionally, it contains other information such as: the different thresholds values and statistics about the fusion. The file also shows a direct link to the OBO-format merged ontology, and a representative image of type of node assignation between ontology A and B (a donut graph) and an image of an Euler-Venn diagram (using squares) about how the fusion has worked.

.html of DAGs and .png of figures files

These files are the files encrusted in the FOntCell_OntologyA_OntologyB.html file.

The .html files are all the different interactive circular graphs, and the .png files contain the Euler-Venn diagram and the donut graph.

Troubleshooting

For parallel computation **FOntCell** requires bigmpi4py:
(https://www.biorxiv.org/content/10.1101/517441v1)

If bigmpi4py has been installed in a different conda environment from the one of the **FOntCell** installation, the parallelization will not work well (all the processes will run on a single processor). In this case, **FOntCell** will work but without parallelization.

Running **FOntCell** will raise a problem if graphviz has not been properly installed. For a correct graphviz installation, type in the command line:

```
sudo apt-get install graphviz
```

Example of how to run FOntCell

Open python3 in bash:

```
sudo python3
```

Import the **FOntCell** module:

import FOntCell

```
import FOntCell
```

After the **FOntCell** module is imported, one can use the following functions:

To run the fusion of two ontologies:

```
FOntCell.run('path_to/configuration_file.txt')
```

To run the demo of the fusion of CELDA with Lifemap:

```
FOntCell.run_demo()
```

To clean internal files from old runs (recommended for use before a new fusion, especially if one of the ontologies from a previous fusion will be used again):

```
FOntCell.clean('path_to/configuration_file.txt')
```

Configuration file for CELDA+LifeMap merging

```
At line start argument must start by '>'
>input_folder='path/to/input/directory/'
>output_folder='path/to/output/directory/'

# Parallelization
>parallelization=False
>proc=5

# # Ontology_parse

>parse_ontology1=True
>parse_ontology2=False

# # # Ontology 1 #
>file1='CELDA_import.owl'
>take_synonyms1=True
>filter_by_keywords1='human', 'mouse'
>ontologyName1=None
>synonym_type1='owlNCBITaxon:synonym'
>label_type1='CELDA:commonName xml:lang="en"', 'CELDA:commonName', 'rdfs:label'
>relative_type1='owl:someValuesFrom'
>file_clean_ontology1='file_clean_CELDA.txt'


# # # Ontology 2 #
>file2='LifeMap_parsed.ods'
>take_synonyms2=True
>filter_by_keywords2='human', 'mouse'
>ontologyName2='LifeMap'
>synonym_type2='owlNCBITaxon:synonym'
>label_type2='rdfs:label')
>relative_type2='owl:someValuesFrom'
>file_clean_ontology2=''


# # ontology_fusion
>del_old_trials_files=False
>onto1_fuseclasses=True
>onto2_fuseclasses=True
>onto1_restriction=False
>onto2_restriction=True
>onto1_list_clear='cell', 'cells'
>onto2_list_clear='cell', 'cells'
>semantical=True
>text_process=True
>split_from1=None
>split_since1=None
>split_from2=None
>split_since2=None
>windowsize=4
>namethreshold=0.85
>localnamethreshold=0.7
>structure_threshold=0.0
>structure_method='blondel'
>automatic=True
```

html output file created by FOntCell for the CELDA+LifeMap merging

In the next pages is attached the html file resulting of using **FOntCell** to merge CELDA and LifeMap ontologies to produce comprehensive cell ontology.

FOntCell
Fusion of CELDA and LifeMap

Interactive circular Directed Acyclic Graphs (DAGs) of (a) CELDA, (b) LifeMap and (c) merged ontologies

(a) DAG of CELDA ontology classes (nodes in orange)
(b) DAG of LifeMap ontology classes (nodes in blue)
(c) DAG of the Fused ontology classes (nodes in orange (from CELDA), blue(from LifeMap), red for structure match and green for name match)

The ontology labels associated to the classes appear when hovering over the nodes.

Some nodes may appear overlapping.

Parameters of the FOntCell fusion algorithm

- Name matching threshold Θ_S: 0.85
- structure matching method: cosine
- Local Name matching threshold Θ_{SL}: 0.7
- Structure matching threshold Θ_T: 0.0

Statistics of the input ontologies

- Number of classes of CELDA ontology: 841
- Number of relations between classes of CELDA ontology: 966
- Number of classes of LifeMap ontology: 796
- Number of relations between classes LifeMap ontology: 924

Statistics of the merged ontology

Statistics of the merged by name mapping

- Number of classes with equivalence found in CELDA by name mapping: 512
- Number of classes with equivalence found in LifeMap by name mapping: 204
- Percentage of classes (in relation to the number of classes of CELDA ontology) added to CELDA by name mapping: 60.88%
- Percentage of nodes added to LifeMap (in relation to the number of nodes of LifeMap ontology) by name mapping: 25.63%

Statistics of the fusion by structure mapping

- Number of classes with equivalence found in CELDA by structure mapping: 179
- Number of classes with equivalence found in LifeMap by structure mapping: 52
- Percentage of classes added to CELDA (in relation to the number of classes of CELDA ontology) by structure mapping: 21.28%

- Percentage of classes added to LifeMap (in relation to the number of classes of LifeMap ontology) by structure mapping: 6.53%

Statistics of the fusion of non-matched nodes

- Number of classes in CELDA non-matched in LifeMap: 150
- Percentage of classes in CELDA non-matched in LifeMap (in relation to the number of classes of CELDA ontology): 17.84%
- Number of classes in LifeMap non-matched in CELDA: 540
- Percentage of classes in LifeMap non-matched in CELDA (in relation to the number of classes of LifeMap ontology): 67.84%

Statistics of the fusion by name and structure mapping

- Number of classes added in total (by name mapping and by structure mapping): 567
- Percentage of classes added in total (by name mapping and by structure mapping): 67.42%
- Number of relations between classes added in total (by name mapping and by structure mapping): 890
- Percentage of relations between classes added in total (by name mapping and by structure mapping): 92.13%

Added classes refers to the descendants classes founded on the mapping

Merged ontology in OBO format

Merged ontology from CELDA and LifeMap:
here

Results of the merged ontology

Percentages of contribution of classes to the merged ontology in relation to the classes of each contributing ontology

Outter circle: Numbers and percentages of CELDA

- Blue: Classes with name match: 512, percentage: 60.88
- Green: Classes with structure match: 179, percentage: 21.28
- Orange: Non-matched classes: 150, percentage: 17.84

Inner circle: Numbers and percentages of LifeMap

- Blue: Classes with name match: 204, percentage: 25.63
- Green: Classes with structure match: 52, percentage: 6.53
- Orange: Non-matched classes: 540, percentage: 67.84

Euler-Venn diagram of the classes of CELDA and LifeMap merging

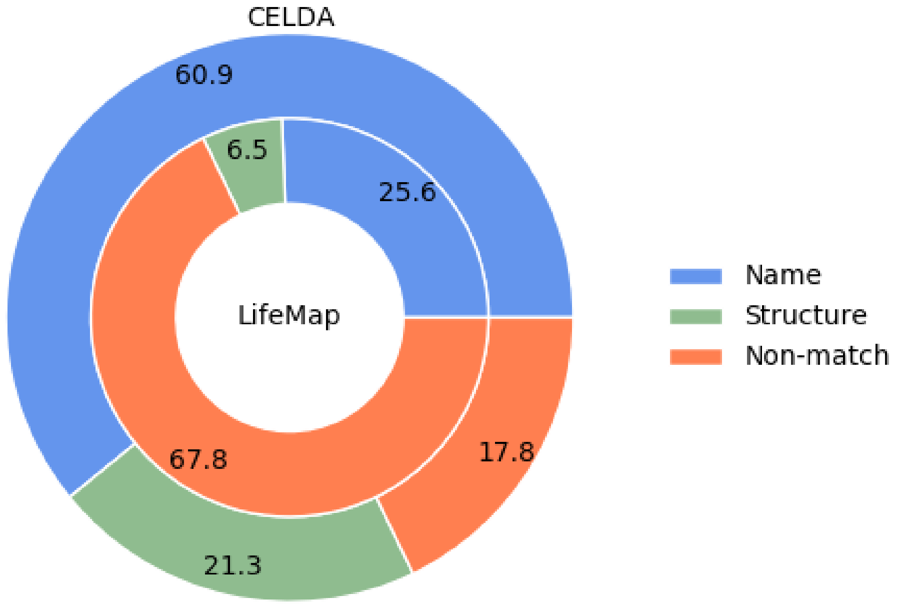

Additional results

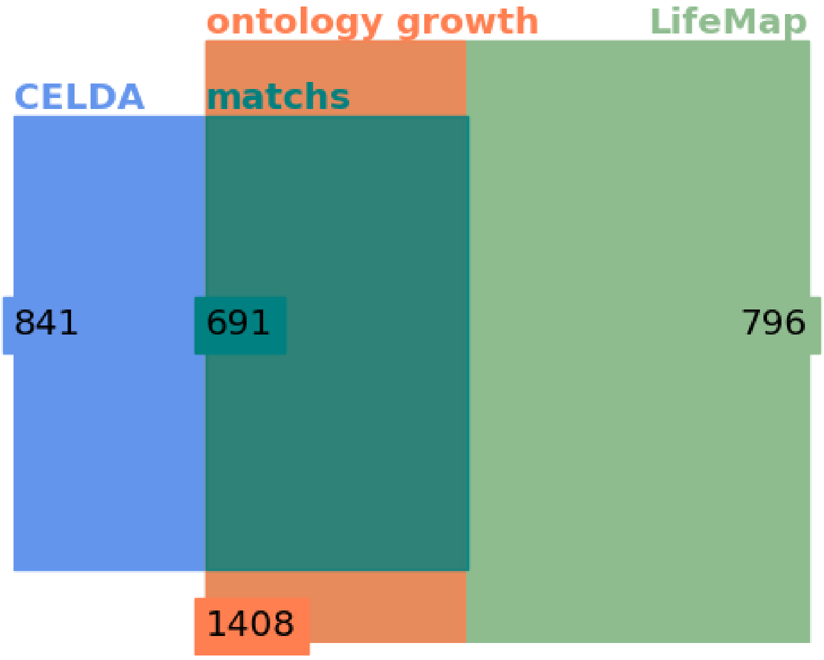

- Classes from CELDA: 841 (blue)
- Classes from LifeMap: 796 (green)
- Classes of LifeMap found to be synonymous of CELDA classes: 691 (blue-green)
- Resulted ontology classes: 1408 (orange)
- Classes added by LifeMap: 1408 – 841 = 567

Files with results on detection of matchs, merging and name matching matrix are available at: /usr/local/lib/python3.6/dist-packages/FOntCell/fontcell_files/

Table S1. Accuracies of CELDA and LifeMap alignment with the different functionalities of FOntCell and the String Equivalence (name matching) method.

	Precision	Recall	F_1	$F_{0.5}$	F_2
**FOntCell (cosine)**	0.877	0.925	0.900	0.886	0.915
**FOntCell (Euclidean)**	0.874	0.915	0.894	0.882	0.906
**FOntCell (Pearson)**	0.877	0.925	0.900	0.886	0.915
**FOntCell (constraint)**	0.847	0.982	0.910	0.871	0.952
**FOntCell (Blondel)**	0.861	0.924	0.891	0.873	0.911
**String Equivalence**	0.986	0.829	0.901	0.950	0.857
