# Supplementary material for: FOntCell: Fusion of Ontologies of Cells
Source: Front Cell Dev Biol. 2021 Feb 11;9:562908. doi: 10.3389/fcell.2021.562908 (PMC7905052; doi:10.3389/fcell.2021.562908)
Supplement: Supplementary file 1 [file Data_Sheet_1.ZIP › AdditionalRawFiles/AdditionalRawFiles/Outputs/CELDA+LifeMap/FOntCell_CELDA_LifeMap.html]

 

# FOntCell Fusion of CELDA and LifeMap

  
  
  
  
  

## Interactive circular Directed Acyclic Graphs (DAGs) of (a) CELDA, (b) LifeMap and (c) merged ontologies

(a) DAG of CELDA ontology classes (nodes in orange)  
(b) DAG of LifeMap ontology classes (nodes in blue)  
(c) DAG of the Fused ontology classes (nodes in orange (from CELDA), blue(from LifeMap), red for structure match and green for name match)  

The ontology labels associated to the classes appear when hovering over the nodes.

Some nodes may appear overlapping.

### Parameters of the FOntCell fusion algorithm

- Name matching threshold ΘS: 0.85
- structure matching method: Blondel
- Local Name matching threshold ΘSL: 0.7
- Structure matching threshold ΘT: 0.0

### Statistics of the input ontologies

- Number of classes of CELDA ontology: 841
- Number of relations between classes of CELDA ontology: 966
- Number of classes of LifeMap ontology: 796
- Number of relations between classes LifeMap ontology: 924

### Statistics of the merged ontology

#### Statistics of the merged by name mapping

- Number of classes with equivalence found in CELDA by name mapping: 512
- Number of classes with equivalence found in LifeMap by name mapping: 204
- Percentage of classes (in relation to the number of classes of CELDA ontology) added to CELDA by name mapping: 60.88%
- Percentage of nodes added to LifeMap (in relation to the number of nodes of LifeMap ontology) by name mapping: 25.63%

#### Statistics of the fusion by structure mapping

- Number of classes with equivalence found in CELDA by structure mapping: 179
- Number of classes with equivalence found in LifeMap by structure mapping: 63
- Percentage of classes added to CELDA (in relation to the number of classes of CELDA ontology) by structure mapping: 21.28%
- Percentage of classes added to LifeMap (in relation to the number of classes of LifeMap ontology) by structure mapping: 7.91%

#### Statistics of the fusion of non-matched nodes

- Number of classes in CELDA non-matched in LifeMap: 150
- Percentage of classes in CELDA non-matched in LifeMap (in relation to the number of classes of CELDA ontology): 17.84%
- Number of classes in LifeMap non-matched in CELDA: 529
- Percentage of classes in LifeMap non-matched in CELDA (in relation to the number of classes of LifeMap ontology): 66.46%

#### Statistics of the fusion by name and structure mapping

- Number of classes added in total (by name mapping and by structure mapping): 547
- Percentage of classes added in total (by name mapping and by structure mapping): 65.04%
- Number of relations between classes added in total (by name mapping and by structure mapping): 886
- Percentage of relations between classes added in total (by name mapping and by structure mapping): 91.72%

Added classes refers to the descendants classes founded on the mapping

  

### Merged ontology in OBO format

Merged ontology from CELDA and LifeMap:

here

## Results on the merged ontology

#### Percentages of contribution of classes to the merged ontology in relation to the classes of each contributant ontology

##### Outter circle: Numbers and percentages of CELDA

- Blue: Classes with name match: 512, percentage: 60.88
- Green: Classes with structure match: 179, percentage: 21.28
- Orange: Non-matched classes: 150, percentage: 17.84

##### Inner circle: Numbers and percentages of LifeMap

- Blue: Classes with name match: 204, percentage: 25.63
- Green: Classes with structure match: 63, percentage: 7.91
- Orange: Non-matched classes: 529, percentage: 66.46

#### Euler-Venn diagram of the classes of CELDA and LifeMap merging

- Classes from CELDA: 841 (blue)
- Classes from LifeMap: 796 (green)
- Synonyms found in CELDA: 691 (Blue-green)
- Resulted ontology classes: CELDA classes: 841 + added classes: 547

## Additional results

Files with results on detection of matchs, merging and name matching matrix are available at: /usr/local/lib/python3.6/dist-packages/FOntCell/fontcell\_files/
