# Supplementary material for: FOntCell: Fusion of Ontologies of Cells
Source: Front Cell Dev Biol. 2021 Feb 11;9:562908. doi: 10.3389/fcell.2021.562908 (PMC7905052; doi:10.3389/fcell.2021.562908)
Supplement: Supplementary file 1 [file Data_Sheet_1.ZIP › AdditionalRawFiles/AdditionalRawFiles/Outputs/CELDA+LifeMap+LMHA/FOntCell_result_LMHA.html]

 

# FOntCell Fusion of result and LMHA

  
  
  
  
  

## Interactive circular Directed Acyclic Graphs (DAGs) of (a) result, (b) LMHA and (c) Fused ontologies

(a) DAG of result ontology (nodes in orange)  
(b) DAG of LMHA ontology (nodes in blue)  
(c) DAG of the Fused ontology (nodes in orange and blue)  

The ontology labels associated to the nodes appear when hovering over the nodes.

Some nodes may appear overlapping.

### Parameters of the FOntCell fusion algorithm

- Sequence similarity threshold ΘS: 0.85
- Topology metric: cosine
- Sequence local similarity threshold ΘSL: 0.7
- Topological similarity threshold ΘT: 0.0

### Statistics of the input ontologies

- Number of nodes of result ontology: 1408
- Number of edges of result ontology: 1855
- Number of nodes of LMHA ontology: 45
- Number of edges of LMHA ontology: 65

### Statistics of the fused ontology

#### Statistics of the fusion by sequence similarity

- Number of nodes with equivalence found in result by sequence similarity: 33
- Number of nodes with equivalence found in LMHA by sequence similarity: 15
- Percentage of nodes (in relation to the number of nodes of result ontology) added to result by sequence similarity: 2.34%
- Percentage of nodes added to LMHA (in relation to the number of nodes of LMHA ontology) by sequence similarity: 33.33%

#### Statistics of the fusion by topological similarity

- Number of nodes with equivalence found in result by topological similarity: 12
- Number of nodes with equivalence found in LMHA by topological similarity: 3
- Percentage of nodes added to result (in relation to the number of nodes of result ontology) by topology similarity: 0.85%
- Percentage of nodes added to LMHA (in relation to the number of nodes of LMHA ontology) by topology similarity: 6.67%

#### Statistics of the fusion of non-matched nodes

- Number of nodes in result non-matched in LMHA: 1363
- Percentage of nodes in result non-matched in LMHA (in relation to the number of nodes of result ontology): 96.80%
- Number of nodes in LMHA non-matched in result: 27
- Percentage of nodes in LMHA non-matched in result (in relation to the number of nodes of LMHA ontology): 60.00%

#### Statistics of the fusion by sequence and topological similarity

- Number of nodes added in total (by sequence similarity and by topological similarity): 29
- Percentage of nodes added in total (by sequence similarity and by topological similarity): 2.06%
- Number of edges added in total (by sequence similarity and by topological similarity): 64
- Percentage of edges added in total (by sequence similarity and by topological similarity): 3.45%

  

### Fused ontology in OBO format

Fused ontology from result and LMHA:

here

## Results on the fused ontology

Percentages of contribution of nodes to the fused ontology in relation to the nodes of each contributant ontologyEuler-Venn diagram of the nodes of result and LMHA fusion  

## Additional results

Files with results on detection of synonyms, graph expansion and sequence similarity matrix are available at: /usr/local/lib/python3.6/dist-packages/FOntCell/fontcell\_files/
