# Supplementary figures and images for: FOntCell: Fusion of Ontologies of Cells
Source: Front Cell Dev Biol. 2021 Feb 11;9:562908. doi: 10.3389/fcell.2021.562908 (PMC7905052; doi:10.3389/fcell.2021.562908)

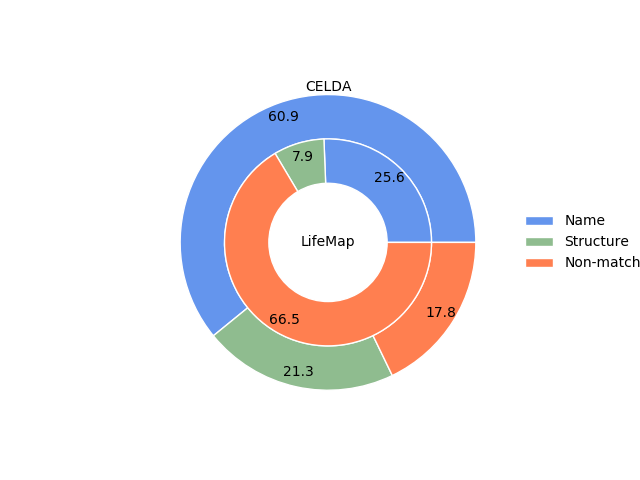

Supplement: Supplementary file 1 [file Data_Sheet_1.ZIP › AdditionalRawFiles/AdditionalRawFiles/Outputs/CELDA+LifeMap/images_and_figures/donut_diagram.png]

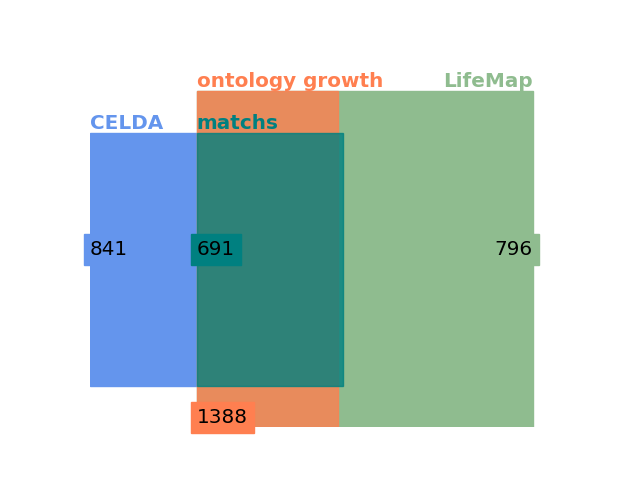

Supplement: Supplementary file 1 [file Data_Sheet_1.ZIP › AdditionalRawFiles/AdditionalRawFiles/Outputs/CELDA+LifeMap/images_and_figures/fusion_square_plot.png]

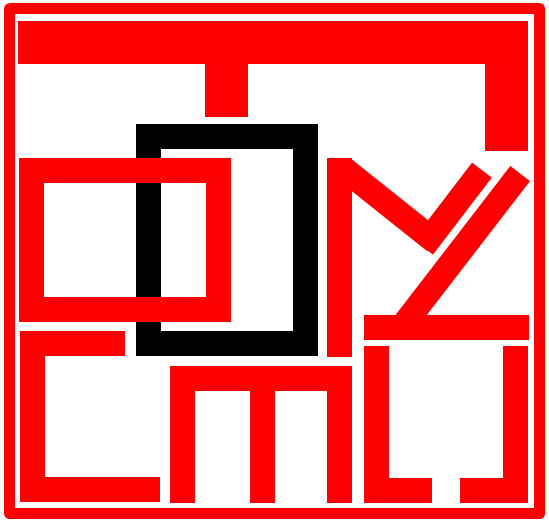

Supplement: Supplementary file 1 [file Data_Sheet_1.ZIP › AdditionalRawFiles/AdditionalRawFiles/Outputs/CELDA+LifeMap/images_and_figures/logo.png]

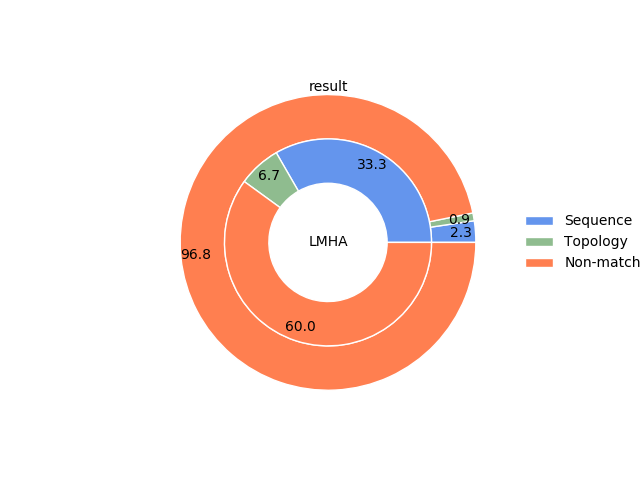

Supplement: Supplementary file 1 [file Data_Sheet_1.ZIP › AdditionalRawFiles/AdditionalRawFiles/Outputs/CELDA+LifeMap+LMHA/images_and_figures/donut_diagram.png]

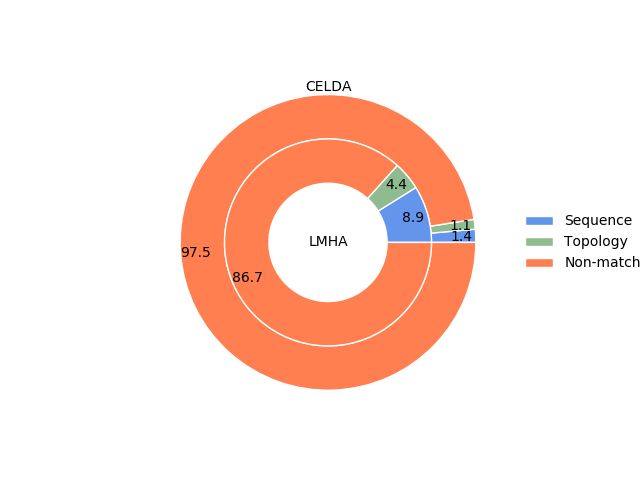

Supplement: Supplementary file 1 [file Data_Sheet_1.ZIP › AdditionalRawFiles/AdditionalRawFiles/Outputs/CELDA+LifeMap+LMHA/images_and_figures/donut_diagram1.png]

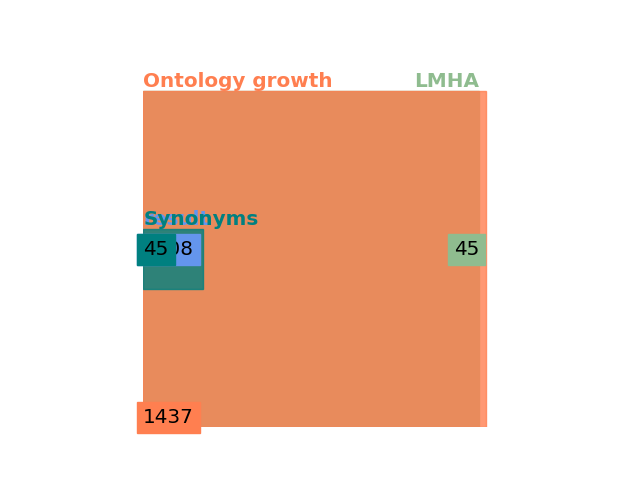

Supplement: Supplementary file 1 [file Data_Sheet_1.ZIP › AdditionalRawFiles/AdditionalRawFiles/Outputs/CELDA+LifeMap+LMHA/images_and_figures/fusion_square_plot.png]
